# Preoperative Inflammatory Blood Indices as Prognostic Markers in Oral Squamous Cell Carcinoma: A Systematic Review and Meta-Analysis

**DOI:** 10.3390/ijms27177638

**Published:** 2026-08-26

**Authors:** Alexandros Louizakis, Dimitris Tatsis, Asterios Antoniou, Ioannis Astreidis, Effimia Stergiadou, Kalliopi Domvri, Konstantinos Paraskevopoulos, Simeon Metallidis, Konstantinos Vahtsevanos, Angeliki Cheva

**Affiliations:** 1 Laboratory of Oral and Maxillofacial Surgery, School of Dentistry, Aristotle University of Thessaloniki, 54124 Thessaloniki, Greece; astrimax23@gmail.com (I.A.); vaxtseva@gmail.com (K.V.); 2 Department of Oral and Maxillofacial Surgery, Aristotle University of Thessaloniki, 54124 Thessaloniki, Greece; dimitats@auth.gr (D.T.); aantoniaf@auth.gr (A.A.); kostparas@yahoo.gr (K.P.); 3 Department of Oral Medicine and Pathology, School of Dentistry, Aristotle University of Thessaloniki, 54124 Thessaloniki, Greece; stergiefi@gmail.com; 4 Department of Pathology, George Papanikolaou General Hospital of Thessaloniki, 54124 Thessaloniki, Greece; kellybio4@hotmail.com; 5 Department of Internal Medicine, AHEPA University General Hospital, Aristotle University of Thessaloniki, 54636 Thessaloniki, Greece; metallidissimeon@yahoo.gr; 6 Department of Pathology, Faculty of Medicine, Aristotle University of Thessaloniki, 54124 Thessaloniki, Greece; antacheva@yahoo.gr

**Keywords:** oral squamous cell carcinoma, neutrophil-to-lymphocyte ratio, lymphocyte-to-monocyte ratio, platelet-to-lymphocyte ratio, prognosis, survival, hematologic parameters, prognostic biomarkers

## Abstract

Oral squamous cell carcinoma (OSCC) prognosis remains limited by TNM staging alone. We conducted a systematic review and meta-analysis to evaluate preoperative neutrophil-to-lymphocyte (NLR), platelet-to-lymphocyte (PLR) and lymphocyte-to-monocyte (LMR) ratios as prognostic markers. We searched PubMed/MEDLINE, Scopus, Base, Google Scholar and ScienceDirect from inception to 30 May 2026. Eligible studies reported preoperative ratios and survival outcomes, namely, overall survival (OS), disease-specific survival (DSS) and disease-free survival (DFS), in surgically treated OSCC. Risk of bias was assessed with the QUIPS tool. Random-effects meta-analyses of multivariate hazard ratios (HRs) were primary; univariate data, heterogeneity, prediction intervals, sensitivity and subgroup analyses were also performed. Thirty-nine studies (12,153 patients; mostly East Asian retrospective cohorts) were included. Elevated NLR predicted worse overall survival (HR 1.51, 95% CI 1.32–1.73) and disease-specific survival (HR 2.00, 1.58–2.54). NLR also showed a significant association with DFS (HR = 1.47, 95% CI: 1.21–1.78), though the prediction interval crossed unity. PLR and LMR showed weaker, less consistent associations. Evidence was limited by predominant retrospective design, geographic concentration and variable cut-offs. Preoperative NLR is a reproducible, inexpensive prognostic biomarker that may complement TNM staging; standardized thresholds require prospective validation. The review was registered in PROSPERO; no specific funding was received.

## 1. Introduction

Oral squamous cell carcinoma (OSCC) is the most common malignancy of the oral cavity and the predominant histological subtype of oral cancer, accounting for more than 90% of cases [1,2,3,4]. On an annual basis, there is a rise in the incidence of OSCC worldwide, reaching approximately 377,000 to 389,000 new cases and nearly 188,000 deaths [4,5,6]. Despite significant advancements in surgical techniques, radiotherapy and the introduction of immunotherapeutic approaches, the prognosis for OSCC patients still remains largely unsatisfactory, with 5-year survival rates ranging between 50% and 60%, suggesting that it has not yet significantly improved in the last decade [4,7,8,9,10,11,12,13,14]. Traditionally, the Tumor-Node-Metastasis (TNM) staging system has been the cornerstone of prognostic assessment and treatment planning for patients with oral cancer; however, this model is anatomically based and does not incorporate other biological factors such as the host immune response or the biological heterogeneity of the tumor. This would merely explain the different outcomes and the variability among patients suffering from the same TNM disease stage [4,15,16,17].

However, the impressive amount of data derived from molecular analyses has changed the limitations of the anatomic-based TNM staging system. Recently, a relationship has been found between systemic inflammation and the presence and stage of oral cancer [4,15,18]. Inflammation has become a well-established hallmark of cancer, contributing significantly to various stages of cancer development, namely, initiation, progression, cancer invasion and metastasis [4,15,18]. In the tumor microenvironment, neutrophils and platelets may promote tumor growth, angiogenesis, and metastasis through the release of cytokines and growth factors such as VEGF, IL-6, and TGF-β, as well as by shielding circulating tumor cells from immune surveillance [2,16,19,20]. By contrast, lymphocytes play a central role in adaptive anti-tumor immunity, and reduced lymphocyte counts may reflect inadequate host immune defense.

This state also triggers systemic inflammatory alterations that are detectable in the peripheral blood [4,20]. As a result, routine preoperative hematological parameters, namely, neutrophils, lymphocytes, monocytes, and platelets, as well as their ratios, namely, the neutrophil-to-lymphocyte ratio (NLR), the lymphocyte-to-monocyte ratio (LMR) and the platelet-to-lymphocyte ratio (PLR), have attracted increasing interest because they are easily accessible and inexpensive and can be used as reproducible biomarkers with potential prognostic value [4,18,21,22,23]. Elevated NLR may indicate an impaired lymphocyte-dependent immune defense, together with increased neutrophil-mediated tumor-promoting activity [1,2,15,16]. Similarly, PLR has been associated with thrombocytosis-related tumor progression and relative lymphopenia, whereas LMR may reflect the interaction between host immune defense and monocyte-derived tumor-associated macrophage activity [2,4,5,18,20,24].

According to the literature, elevated NLR and PLR values in the peripheral blood are strongly associated with advanced stages of oral cancer and sometimes with faster progression, along with perineural invasion (PNI), positive cervical lymph nodes and extracapsular lymph node extension (ENE) [9,16,21]. Finally, a correlation has been observed, with increased likelihood of recurrence and reduced disease-free survival (DFS), overall survival (OS) and disease-specific survival (DSS) [9,16,21]. To continue, some studies have identified NLR as the most robust predictor of OS, whereas others have suggested that PLR may be more informative when it comes to DFS; similarly, the prognostic relevance of LMR is yet to be confirmed across cohorts [4,16,20,24].

These changes in blood cell counts are not random—they trace back to the systemic inflammation that tumors generate as they progress. Neutrophils and platelets assist tumors by promoting the growth of new blood vessels and shielding circulating tumor cells from immune attack, while a decline in lymphocytes is a sign that a tumor is actively suppressing the body’s own defenses [16,19,25]. Monocytes also play a role, frequently differentiating into tumor-associated macrophages that promote angiogenesis and invasion, which is one reason why LMR may have prognostic significance [26,27].

These ratios are not entirely cancer-specific, though, as corticosteroid use, infection, smoking, and other baseline population differences can also alter their figures significantly [28,29]. Such biological variability, together with etiologic heterogeneity in OSCC, likely also contributes to the inconsistent cut-off values reported across studies and supports the need for a pooled meta-analytic approach [30,31].

In addition, other important biomarkers also highlight the biological heterogeneity in the disease beyond anatomical staging alone [4,7,15,29,32]. These include p53, COX-2, and PD-L1, as well as circulating markers such as microRNAs and serum proteins [3,19,29,33,34]. All the above have contributed to the evolution of cancer. However, the routine clinical use of many of these molecular biomarkers remains limited by cost; they can be technically complex, with the need for specialized tissue-based analysis, and they may sometimes lack standardization. On the other hand, peripheral blood inflammatory markers such as NLR, PLR, and LMR offer a more accessible and easily reproducible approach when it comes to prognostic stratification [3,4,22,29].

However, although numerous studies have evaluated the prognostic values of these biomarkers, inconsistency persists among the available evidence [5,18,35]. Uncertainty remains regarding their independent predictive value, and no clear consensus has yet been established on the optimal cut-off values for the clinical application of the above markers [5,22]. Accordingly, this systematic review and meta-analysis aims to evaluate the prognostic value of preoperative NLR, PLR, and LMR in patients with OSCC. Specifically, its main objective is to shed some light on the associations of these biomarkers with OS, DSS, and DFS and also to assess their potential role in prognostic stratification and personalized therapeutic management of OSCC patients [7,14,25,26,27,28,29,31,36,37,38,39].

In addition, because these indices have increasingly been explored as components of multivariable prognostic models and nomograms [1,2,3], clarifying their independent and reproducible prognostic contribution may also help define their future role in individualized surveillance and treatment intensity planning [1,2,3,4,5,6].

## 2. Materials and Methods

### 2.1. Information Sources and Search Strategy

This meta-analysis was conducted in accordance with the PRISMA 2020 statement and the AMSTAR guidelines for assessing the methodological quality of systematic reviews. The study protocol was prospectively registered in PROSPERO. The completed PRISMA 2020 checklists for the abstract and the main manuscript are provided in Supplementary Tables S2 and S3, respectively.

Two investigators independently performed literature searches in [PubMed/MEDLINE], [Scopus], [Base], [Google Scholar] and [ScienceDirect] from database inception to 30 April 2026. The search strategy combined three major concept groups using both MeSH terms and free-text keywords: peripheral inflammatory blood markers, including NLR, PLR, and LMR; oral cancer-related terms, including relevant anatomical subsites and carcinoma descriptors; and survival outcomes, with particular emphasis on overall survival (OS), disease-specific survival (DSS), and disease-free survival (DFS). The complete search strategy is provided in Supplementary Table S1. The search strategy was supplemented by manual screening of the reference lists of related articles.

### 2.2. Selection Process

**Figure 1:**
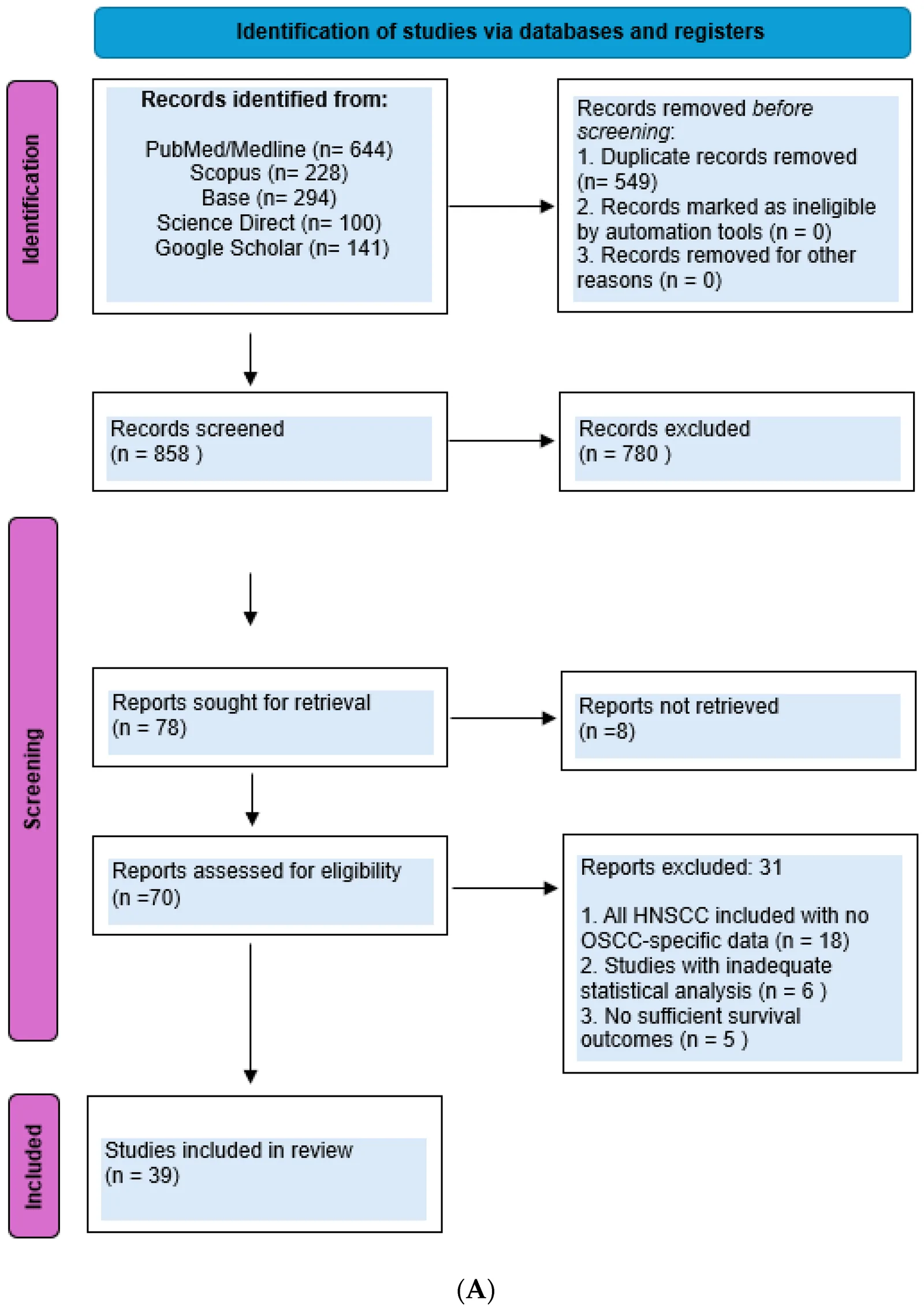

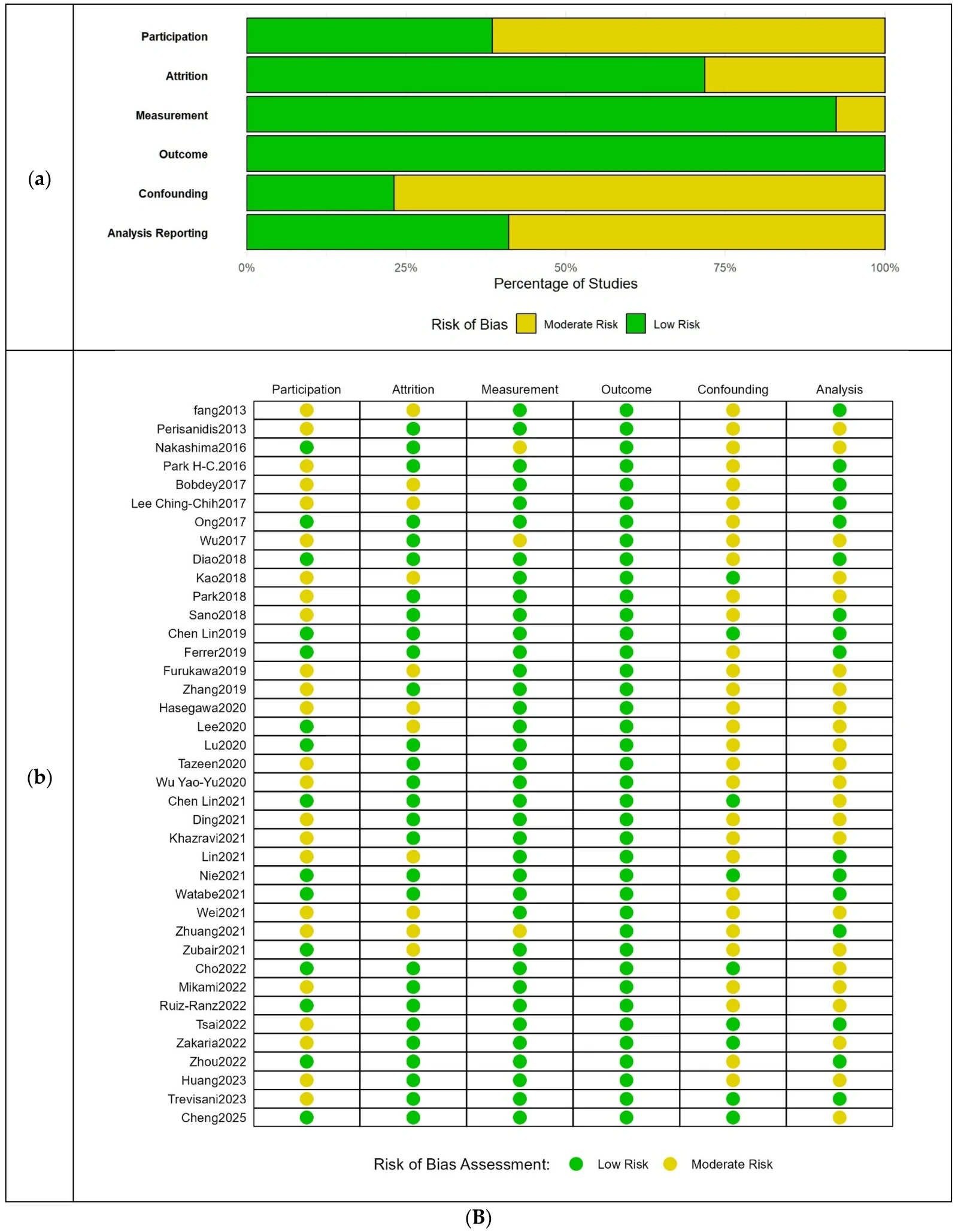
(**A**) PRISMA 2020 flow diagram for the systematic review, which included searches of databases, registers and other sources. (**B**) Risk of bias: (**a**) distribution of low-, moderate- and high-risk (0%) studies in each domain; (**b**) characterization of each one of the included studies across the six domains.

Two reviewers independently conducted study selection in two sequential stages: (1) title and abstract screening to remove clearly irrelevant records, followed by (2) full-text assessment to determine final eligibility. The review was restricted to primary squamous cell carcinomas of the oral cavity. Studies predominantly involving oropharyngeal or other non-oral head and neck subsites were excluded unless OSCC-specific data could be extracted separately. During the initial screening, studies were considered eligible if they referred to oral squamous cell carcinoma (OSCC) and at least one of the biomarkers of interest. Records were excluded for the following reasons: (1) they were not related to head and neck malignancies, (2) they did not address prognostic factors (e.g., studies focused on treatment interventions, basic science, or unrelated biomarkers), (3) records of peripheral blood inflammation markers were not available preoperatively and (4) patients received non-surgical treatments, such as radiotherapy and/or chemotherapy, instead of surgery. Conference abstracts were excluded unless a full-text version was available. For studies assessed at the full-text stage, reasons for exclusion were systematically recorded (e.g., inappropriate patient population, absence of survival or prognostic outcomes, or lack of extractable data). Any disagreements between reviewers were resolved through discussion and consensus, with consultation from a senior reviewer when necessary. The overall selection process is summarized in the PRISMA flow diagram provided below (Figure 1A).

### 2.3. Statistical Methods

To ensure a standardized direction of effect across all included studies, hazard ratios (HRs) originally reporting the reference standard as low vs. high were mathematically inverted (1/HR) to a uniform high vs. low comparison. Consequently, regarding marker LMR—and consistent with the analyses for NLR and PLR—effect sizes plotted to the right of the vertical null axis (HR > 1.0) strictly indicate that a low LMR is significantly associated with reduced overall survival (OS). Where primary studies did not report univariate hazard ratios, missing data were estimated utilizing the established methodologies described by Tierney et al. (2025) [37]. Following the methodological framework outlined by Riley et al. (2019) [38], it is imperative to evaluate a biomarker’s independent prognostic value over and above existing clinical parameters. Therefore, separate meta-analyses were conducted: adjusted HRs from multivariate analyses constituted the primary analysis, while unadjusted HRs from univariate analyses served as the secondary analysis, with the latter primarily utilized to explore between-study heterogeneity. Data synthesis was initially conducted utilizing a DerSimonian and Laird (DL) random-effects model [39] with the Knapp–Hartung variance adjustment [40], while the Jackson method [41] was used to calculate exact confidence intervals regarding the between-study variance (tau-squared and tau). I-squared was used to estimate the statistically defined heterogeneity among studies (25%, 50%, and 75% considered as low, moderate, and high, respectively [42]). The fixed-effects model was applied only in instances of extreme homogeneity, where the DL estimator was set as tau-squared equals zero. For all pooled outcomes, 95% confidence intervals (CIs) were reported to describe the precision of the mean effect, alongside 95% prediction intervals (PIs) to evaluate the distribution of true effect sizes and hence the robustness of the prognostic effect in future clinical settings under the presence of heterogeneity [38,43]. In all forest plots, the expected HR estimate (point estimate) is shown with a black diamond, having width equal to the range of the 95% CI., while the 95% prediction interval is represented by a red line segment. Further robustness of the pooled estimates was tested through leave-one-out sensitivity analyses and temporal cumulative meta-analyses sorted by publication year. For analyses encompassing 10 or more independent cohorts, the presence of small-study effects and potential publication bias was assessed through visual inspection of contour-enhanced funnel plots and radial plots, followed by formal statistical testing via Egger’s continuous linear regression [44]. In the presence of detectable bias, effect size adjustments were computed utilizing the non-parametric trim-and-fill method [45] for small-study effects and the Copas selection model [46] for selection bias. Subgroup analyses were conducted to investigate potential sources of population heterogeneity. Due to the limited number of studies per stratum, year of publication was dichotomized according to cumulative meta-analysis on ascending years, while other continuous covariates were dichotomized at their respective overall medians prior to subgrouping. The association between the applied diagnostic cut-off thresholds and the log hazard ratios was studied by inspection of a scatterplot and cumulative meta-analysis of descending thresholds. All statistical and graphical computations were executed in the R statistical language, version 4.4.2 [47], in the RStudio IDE (RStudio version 2025.09.01) [48] utilizing the ‘meta’ [49] and ‘metafor’ libraries [50]. A two-sided *p*-value of < 0.05 was considered statistically significant for all tests.

### 2.4. Characteristics of the Included Studies and Risk of Bias Analysis

Of the 1407 studies identified, a total of 39 independent studies, published between 2013 and 2025, met the eligibility criteria and were included in the final meta-analysis [1,2,3,5,8,9,10,12,13,14,15,16,17,18,19,20,21,24,25,26,27,28,29,31,32,35,36,51,52,53,54,55,56,57,58,59,60,61,62]. Duplicates were removed by importing all citations into a reference manager (Endnote). After duplicate removal, titles and abstracts were screened independently by two reviewers, followed by full-text assessment of potentially eligible studies. The search strategy was adapted to the indexing system and syntax of each database, with broader keyword combinations used in Google Scholar and more structured controlled vocabulary and field-specific searching used in PubMed and Embase (Figure 1A).

All but the large-scale prospective study by Zhuang et al. (2021) [25] included retrospective cohorts. A total of 12,153 patients were recruited; sample sizes ranged from 40 to 890, with a median of 226 patients from two geographic regions: ‘East Asian’ and ‘Other’. Most of the study populations, amounting to 79.5%, were predominantly derived from studies in East Asia, with 9534 patients in total from 13 studies in China, 7 in Taiwan, 6 in Japan, 3 in the Republic of Korea and 1 each in Malaysia and Korea. The category ‘Other’ comprised 2619 patients from 2 studies each in Spain and India and 1 each in Austria, Brazil, Iran and the UK. In the total cohort, the median age ranged from 39.9 to 71 years (median: 60); the percentage of males ranged from 8.4% to 93%, median 63%; and the percentage of patients with tumor Stage I or II ranged from 0% to 100%, median 41%. Tongue was the most frequently implicated anatomical subsite, ranging from 17% to 100%, median = 41% (found in 30 studies), and Buccal–Mucosa (given in 23 studies) ranged from 2% to 45%, median 14%. Ging_Alveol was given in 16 studies and ranged from 10% to 35%, median 17%. Floor was given in 18 studies and ranged from 1% to 30%, median 7%. Hard_P was given in 12 studies and ranged from 2% to 15%, median 5.5%. Lip was given in 9 studies and ranged from 1% to 8%, median 2%. Retr_Tr was given in 9 studies and ranged from 1% to 38%, median 6%. Other Sites was found in 11 studies and ranged from 0.6% to 51%, median 17%. Cases (deaths or recurrences) per study were given in 33 studies and ranged from 6% to 60%, median 23%. Regarding major confounders that play crucial roles in marker studies, of the 14 defined in our study to be the minimum needed, their number ranged from 3 (21%) to 10 (71.4%), with a median of 5 (36%). The complete list and details of the 39 eligible studies are summarized in Table 1.

The Quality in Prognosis Studies (QUIPS) tool was used to evaluate the risk of bias (RoB) for the 39 included studies, aligning with the methodological framework for prognostic factor meta-analyses outlined by Riley et al. (2019) [39]. Overall, the methodological quality of the included literature was acceptable, with no studies exhibiting a ‘high risk’ of bias across any of the six evaluated domains (Figure 1B(a,b)). The cohorts demonstrated exceptional rigor in the Outcome Measurement domain, with 100% of the studies rated as low risk, indicating standardized and reliable assessment of survival endpoints. Similarly, Measurement of the Prognostic Factor and Study Attrition performed strongly, with approximately 90% and 75% of the studies achieving a low-risk rating, respectively. Conversely, the most substantial methodological limitations were observed in the Study Confounding domain, where roughly 75% of the studies were of moderate risk. This high prevalence of moderate risk indicates significant heterogeneity in how primary authors selected and adjusted for critical clinical covariates. Furthermore, both Study Participation and Statistical Analysis and Reporting exhibited moderate risk in approximately 60% of the studies (attributed to limitations in retrospective prognostic research).

## 3. Results

### 3.1. NLR Marker

A total of 36 independent studies evaluated the prognostic value of the NLR marker (all but one, that by Zhuang et al. 2021, which was also a large-scale prospective study, were retrospective) [25]. Across all clinical endpoints, primary analyses were based on studies reporting complete multivariate hazard ratios (HRs) and 95% confidence intervals (CIs). Secondary analyses (which aimed to show increased heterogeneity in comparison to multivariate results) utilized univariate data, which were either directly reported or estimated using the methods described by Tierney 2025. Regarding each endpoint, for OS, univariate results were reported in 22 studies and were estimated in seven studies (29 studies in total), while multivariate results were given in 20 studies; for DFS, univariate results were reported and estimated in 12 and two studies, respectively (14 studies in total), while multivariate results were given in 11 studies; and, finally, for DSS, univariate results were reported and estimated in five and seven studies, respectively (12 studies), while multivariate results were reported in eight studies.

#### 3.1.1. NLR and OS

The primary meta-analysis of the association between NLR and overall survival (OS) included 20 of the 29 identified studies that reported complete multivariate results. The pooled cohort comprised 7140 patients across six countries. Geographically, 16 studies evaluated East Asian populations (China, Japan, Republic of Korea, and Taiwan), while the remaining four evaluated other populations (India and Spain).

Across the included studies (in the primary analysis), the median sample size was 276.5 patients (range, 94–890), and the median follow-up period was 4 years (range, 2–6 years). The median of the median or mean patient age across the cohorts was 60 years (range of medians, 50–71 years), and the median proportion of male patients per study was 64% (range, 8.4–93.0%). Regarding tumor characteristics, early-stage disease (Stages I or II) accounted for a median of 40% per study (range, 0–100%). The percentage of cases (deaths in OS) was reported or estimated in 18 studies, and the median was 24.5% (range, 8.3–68%).

Tongue was the most frequently involved anatomical subsite and was reported in 16 studies, with a median percentage of 39.5% (range, 17–100%). Buc_Muc was reported in 12 studies, with a median percentage of 15% (range, 7–43%). Ging_Alveol was reported in 10 studies, with a median percentage of 14% (range, 8–35%). Floor was reported in nine studies, with a median percentage of 7% (range, 1–30%). Other_S was reported in seven studies, with a median percentage of 18% (range, 0.6–51%). Hard_P was reported in six studies, with a median percentage of 4.5% (range, 0.4–7%). Retr_Tr was reported in six studies, with a median percentage of 6% (range, 4–38%). Lip was reported in four studies, with a median percentage of 2.5% (range, 2–8%).

Given the observed demographic and clinical differences among the included populations, as well as the fact that over half of the included studies adjusted for fewer than seven of the 14 predefined critical confounders (detailed in the footnote of Table 1), with a median percentage of critical confounders included per study of 35.7% (range, 21–71.4%), a random-effects model was used for the meta-analyses of both univariate and multivariate data.

**Figure 2:**
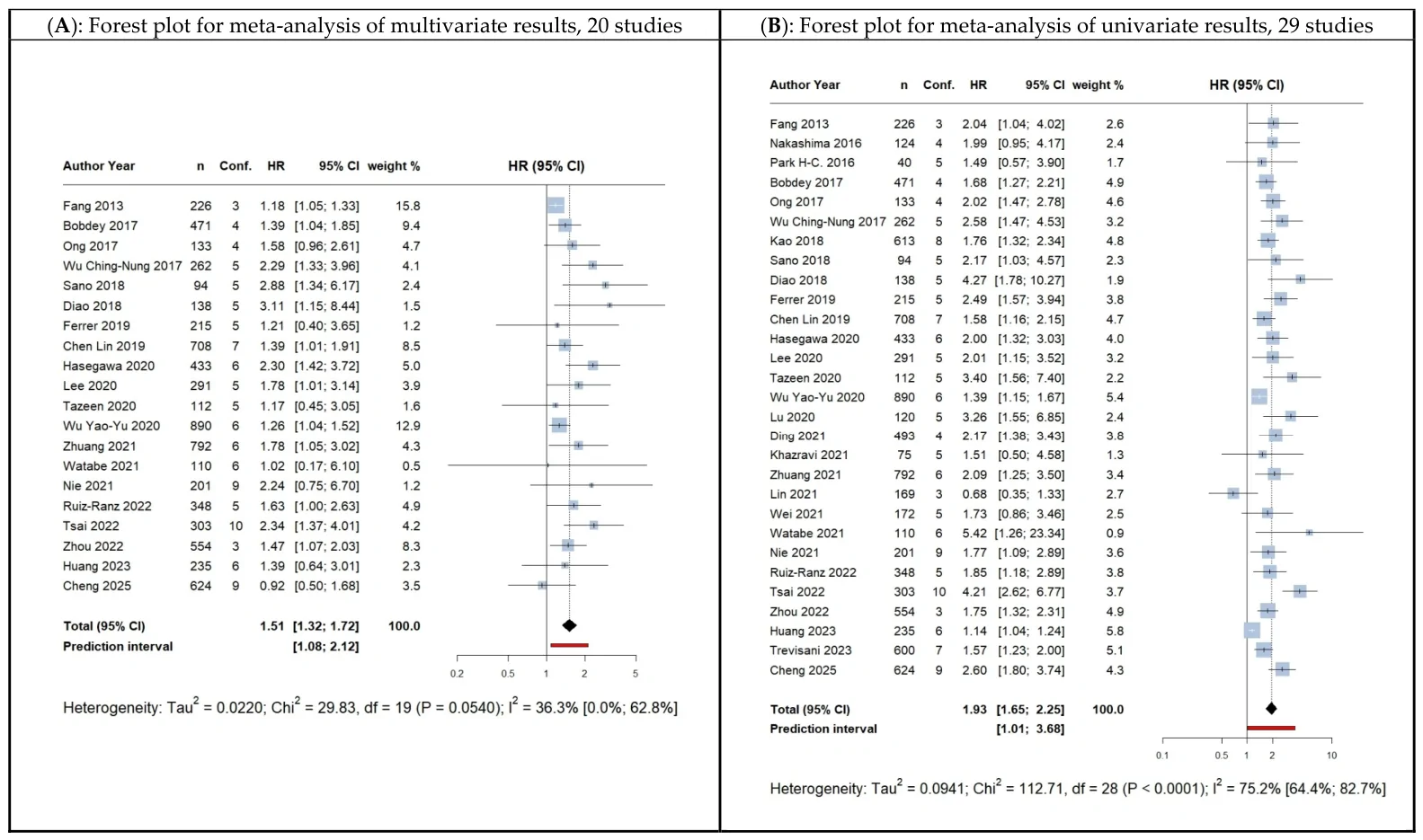
Forest plots for the meta-analysis of NLR and OS: left, (**A**) primary meta-analysis of the multivariate results of 20 studies and, right, (**B**) secondary meta-analysis of the univariate results of 29 studies.

Regarding the association between NLR and overall survival (OS), the secondary analysis of the univariate results from 29 studies [1,2,3,5,8,9,10,13,14,15,16,17,19,20,25,26,27,28,29,35,36,52,54,55,56,59,61] revealed high interstudy heterogeneity, I-squared: 75.2%, 95% CI: 64.4–82.7%, *p* < 0.0001, tau = 0.31, 95% CI: 0.17–0.46 (Figure 2B), underscoring the substantial confounding biases present when adjusting variables are omitted. Conversely, the primary analysis utilizing the adjusted multivariate HRs and 95% CIs from 20 studies demonstrated non-significant low to moderate between-study heterogeneity, I-squared: 36.3%, 95% CI: 0–62.8%, *p* = 0.054, tau = 0.02, 95% CI: 0.0–0.12. Applying a DerSimonian and Laird random-effects model with Knapp–Hartung variance adjustments and the Jackson method for confidence intervals of tau-squared and tau, the pooled effect estimate was HR = 1.51 (95% CI: 1.32 to 1.73), *p* < 0.0001, suggesting that large values of NLR are related to poor OS. Crucially, regarding both analyses (using univariate and multivariate data, Figure 2) the 95% prediction interval (95% PI) ranged from 1.08 to 2.12 in the multivariate case and from 1.01 to 3.68 in the univariate case, demonstrating that high preoperative NLR serves as a robust, independent prognostic factor for poor overall survival. This conclusion is expected to remain statistically significant in future comparable clinical populations.

**Figure 3:**
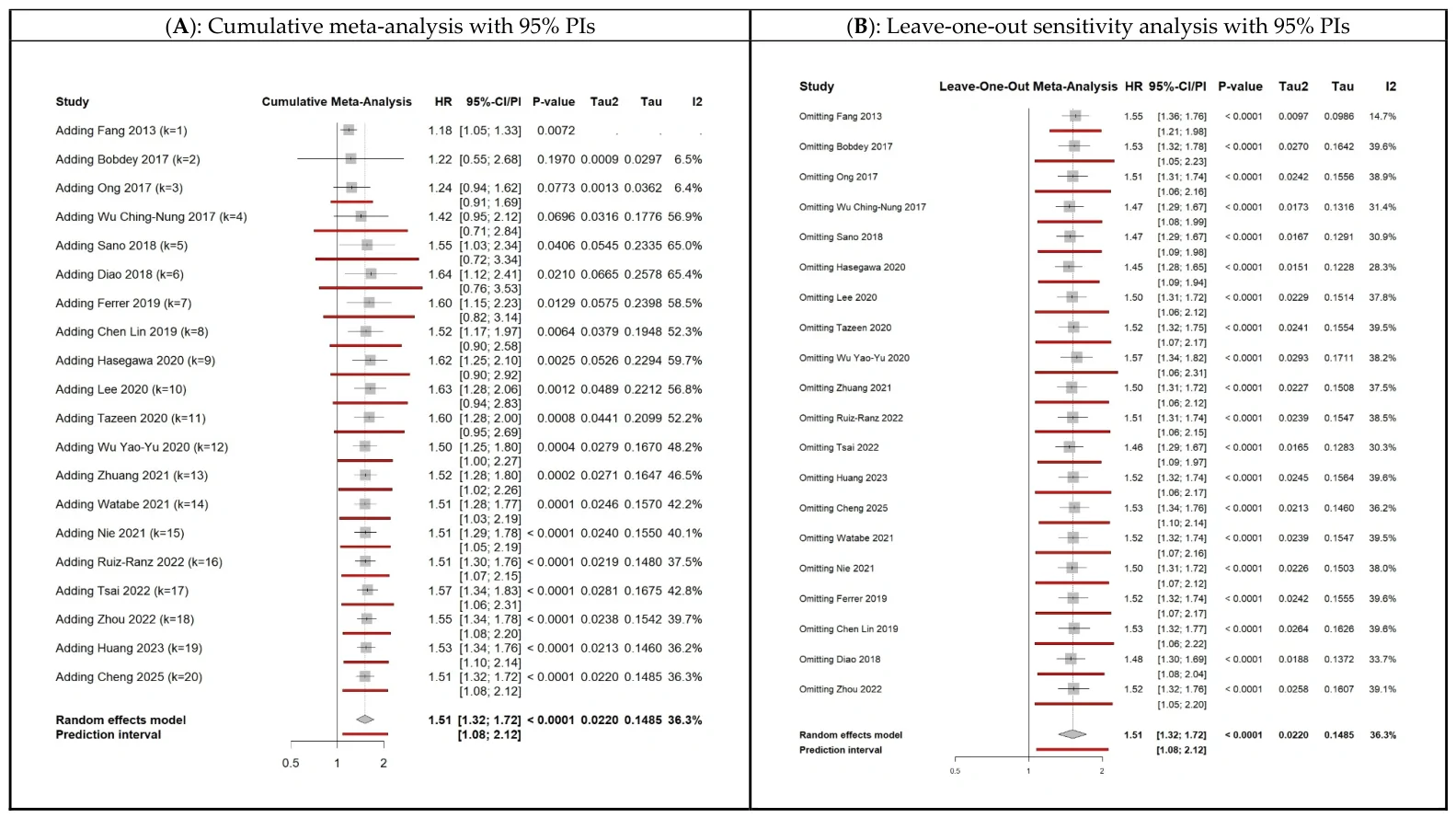
Forest plots: left, (**A**) cumulative meta-analysis of the multivariate data and, right, (**B**) leave-one-out sensitivity analysis for NLR and OS. The plots also include the 95% prediction interval represented by a red linesegment.

To further validate the stability of this primary pooled estimate, a cumulative meta-analysis and a leave-one-out sensitivity analysis were performed. The cumulative meta-analysis, as shown in Figure 3A, indicates the pooled HR achieved sustained statistical significance (lower bound of 95% CI > 1.0) after Sano et al. (2018) [19]. Although the results in the studies by Watabe et al. (2021), Nie et al. (2021), Huang et al. (2023) and Cheng et al. (2025) do not support our findings [17,26,30,52], the 95% PI established predictive significance (lower bound of 95% PI > 1.0) following Zhuang et al. (2021) [25], crucially, as this was the only prospective study in addition to a large scale one including 792 patients. The leave-one-out sensitivity analysis, as depicted in Figure 3B, also confirms model stability; the exclusion of any single study altered neither the statistical significance nor the 95% PI of the overall HR, suggesting that the synthesized findings are not skewed by any individual influential cohort.

**Figure 4:**
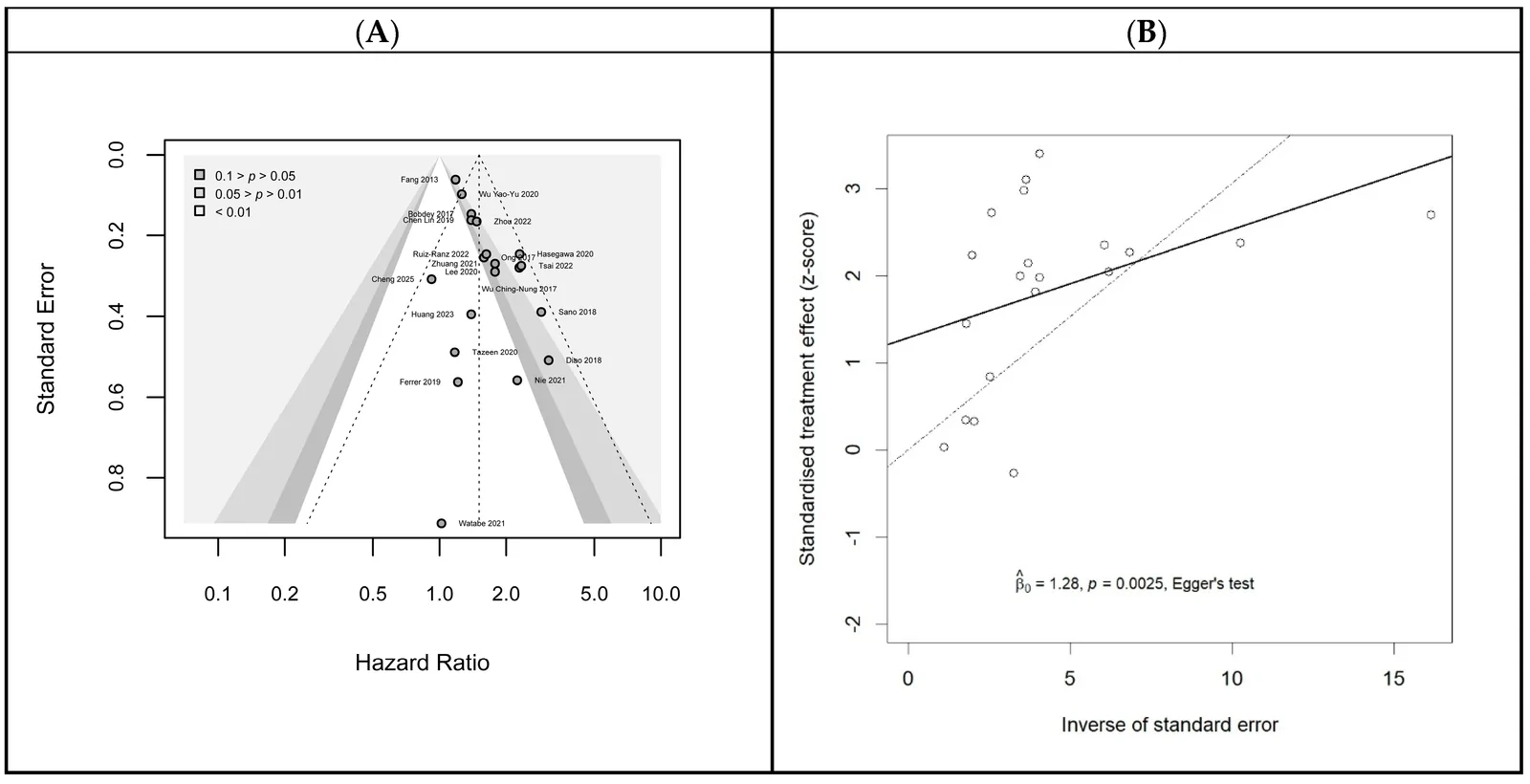
(**A**) Contour-enhanced funnel plot. (**B**) Egger’s linear regression and radial plot (dashed line).

Potential publication bias caused by small-study effects was evaluated via visual inspection of a contour-enhanced funnel plot and Egger’s linear regression test (Figure 4, including the corresponding radial plot). Both assessments revealed statistically significant funnel plot asymmetry. Given the low to moderate between-study heterogeneity, the non-parametric trim-and-fill method and the Copas selection model were employed to calculate an adjusted pooled HR. The trim-and-fill algorithm imputed seven missing studies, yielding an attenuated but significant adjusted HR of 1.32 (95% CI: 1.11–1.56, *p* = 0.003).

Similarly, the Copas selection model estimated nine unpublished studies, resulting in an adjusted HR of 1.26 (95% CI: 1.18–1.35, *p* < 0.0001). Both analyses demonstrate that while publication bias likely inflated the unadjusted pooled estimate, the conclusion about NLR and OS remains stable.

In order to explore other causes of heterogeneity among study populations, subgroup analyses were performed on fully reported (i.e., no missing values) study-level characteristics, including publication year (Year), geographic area (Area), median follow-up duration (Follow-up), sample size, proportion of male patients (Males (%)), and the number of critical confounders (Confounders), as well as the aggregated patient-level characteristic median of patient age (Age). For categorical conversion, publication year was dichotomized at 2021, representing the temporal threshold in cumulative meta-analysis where the lower bound of the 95% prediction interval (PI) permanently stabilized above 1.00. All remaining continuous covariates were dichotomized at their respective sample medians (Table 2). Although non-statistically significant differences were found between subgroups, it is worth noting the very small number of studies (only four) on populations other than East Asian populations, which severely limits the statistical power of this comparison and suggests that marker NLR does not seem to correlate with OS in non-East Asian populations, as shown in Table 2. Furthermore, regarding median age, studies with a median age greater than 60 tend to show greater correlation between NLR and OS (HR = 1.73, 95% CI: 1.35–2.23) than studies with a median age of at most 60 (HR = 1.39, 95% CI: 1.17–1.64), *p* = 0.093 < 0.1.

**Figure 5:**
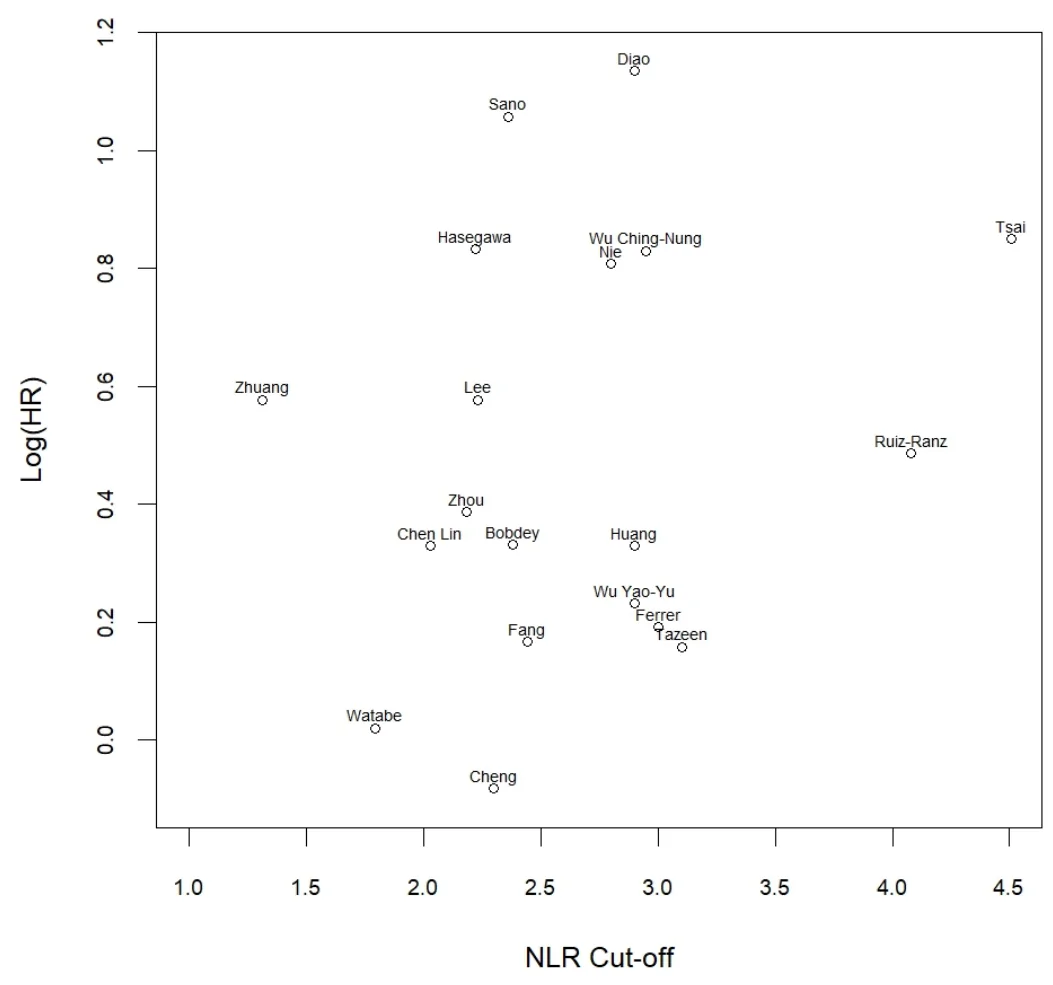
Scatter plot of NLR cut-off values versus log(HR)s for 19 of the 20 included studies.

NLR cut-off thresholds were explicitly reported in 19 of the 20 included studies, ranging from 1.31 to 4.51 (median: 2.44). The authors of one study (Ong et al., 2017) [59] did not define an optimal cut-off, as NLR was not significantly associated with overall survival in their cohort. A non-significant positive linear correlation (r = 0.22, *p* = 0.3752) was observed between the log hazard ratios and the applied cut-off thresholds. Furthermore, visual inspection of the scatter plot (Figure 5) reveals a random distribution of log hazard ratios across these cut-offs, indicating that the magnitude of the prognostic effect does not systematically vary with the chosen threshold but rather may depend on the underlying characteristics of the study populations. However, a cumulative meta-analysis, sorted by ascending cut-off values, demonstrated that the lower bound of the 95% prediction interval (PI) permanently exceeded 1.0 at a threshold of 2.9. Consequently, the sample median (2.44) and the predictive stability threshold (2.90) represent empirically justified starting points for determining optimal cut-offs in future prospective trials.

#### 3.1.2. NLR and DFS

The primary meta-analysis of the association between NLR and DFS included 11 of the 14 identified studies that reported complete multivariate results. The pooled cohort comprised 3000 patients across four countries, all from East Asian populations (China, Japan, Republic of Korea, and Taiwan).

Across the included studies, the median sample size was 235 patients (range, 110–624), and the median follow-up period was 4 years (range, 3–5.5 years). The median of the median or mean patient age across the cohorts was 60 years (range of medians, 51–71 years), and the median proportion of male patients per study was 59% (range, 8.4–90%). Early-stage disease (Stages I or II) was reported in nine studies and accounted for a median of 45% per study (range, 25–100%). The percentage of cases (in DFS) was reported or estimated in 10 studies, and the median was 27.5% (range, 8.3–56%).

**Figure 6:**
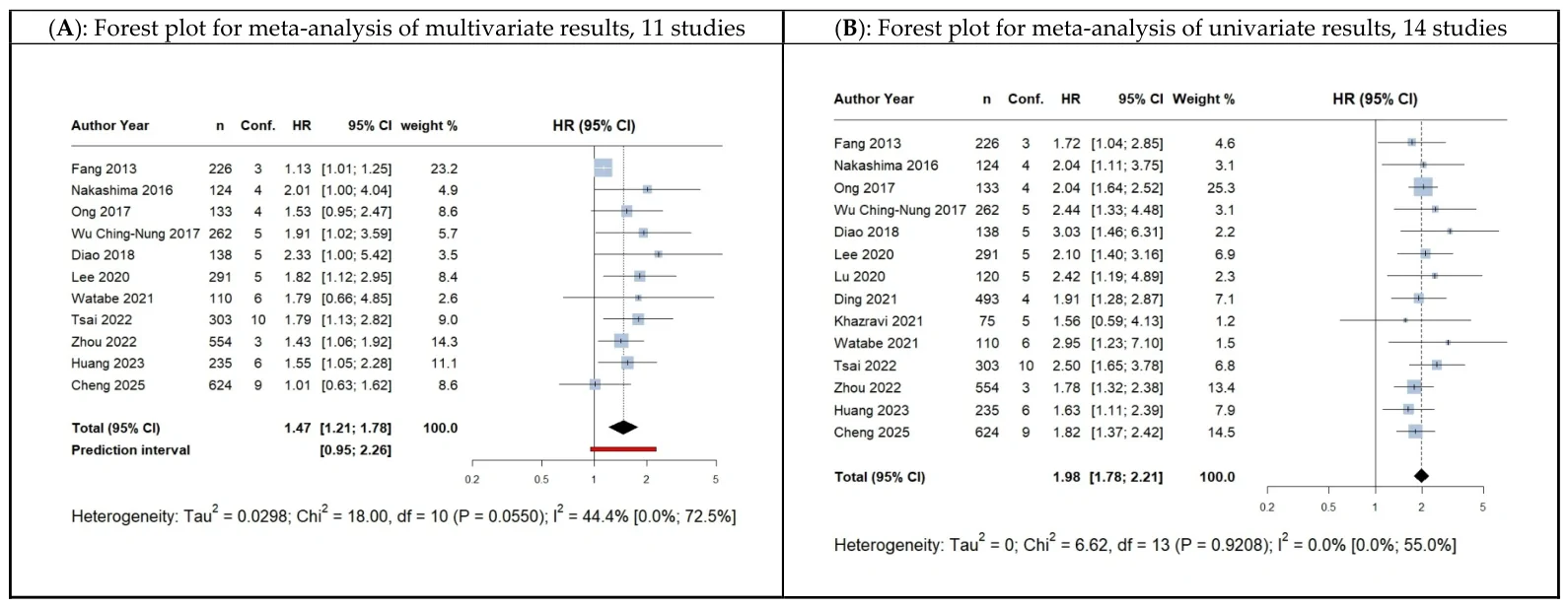
Forest plots for the meta-analysis of NLR and DFS: left, (**A**) primary meta-analysis of the multivariate results of 11 studies and, right, (**B**) secondary meta-analysis of the univariate results of 14 studies.

Tongue was the most frequently involved anatomical subsite and was reported in eight studies, with a median percentage of 39.5% (range, 21–100%). Buc_Muc was reported in six studies, with a median percentage of 21.5% (range, 14–43%). Ging_Alveol was reported in four studies, with a median percentage of 19% (range, 8–29%). Floor was reported in four studies, with a median percentage of 5.5% (range, 4–15%). Other_S was reported in two studies (16% and 18%, median: 17%). Hard_P was reported in four studies, with a median percentage of 4.5% (range, 0.4–18%). Retr_Tr was reported in four studies, with a median percentage of 5.5% (range, 4–12%). Lip was reported in four studies, with a median percentage of 2.5% (range, 2–8%). Regarding critical confounders, their median percentage included per study was 35.7% (range, 21–71.4%). A fixed-effects model was used for the secondary analysis, and a random-effects model was used for the meta-analyses of multivariate data; their results are presented as forest plots in Figure 6, B and A, respectively.

The secondary analysis of the univariate results for DFS [1,3,9,10,13,14,16,17,20,29,35,36,52,54,55] revealed low interstudy heterogeneity, I-squared: 0%, 95% CI: 0–55%, with a zero tau (tau = 0, 95% CI: 0.0–0.14), as shown in Figure 6B; hence, a fixed-effects model was used and no 95% PI was calculated. The pooled effect was HR = 1.98 with a 95% CI of 1.78–2.21, *p* < 0.0001. In contrast, the primary analysis demonstrated low to moderate between-study heterogeneity: I-squared: 44.4%, 95% CI: 0–72.5%, *p* = 0.055, tau = 0.17, 95% CI: 0.0–0.41. Under the random-effects model, the pooled hazard ratio demonstrated a statistically significant association between elevated NLR and reduced DFS: HR = 1.47, 95% CI: 1.21–1.78, *p* = 0.0013. However, the 95% PI of 0.95–2.26 crossed the null value of 1.0; hence, the true prognostic effect of NLR on DFS may not remain significant in future studies.

**Figure 7:**
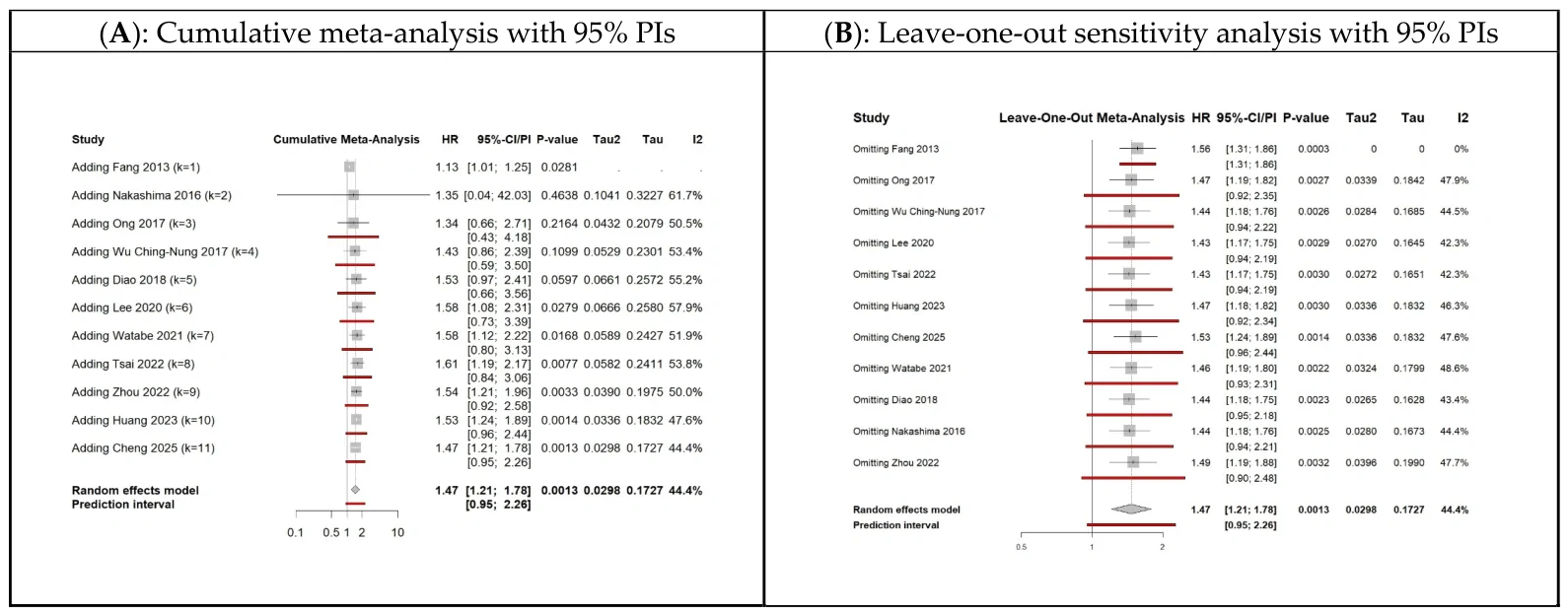
Forest plots: left, (**A**) cumulative meta-analysis of the multivariate data and, right, (**B**) leave-one-out sensitivity analysis for NLR and DFS. The plots also include 95% prediction intervals on the right of the red segments.

The cumulative meta-analysis (Figure 7A) demonstrates that the pooled HR achieved sustained statistical significance following the inclusion of Lee et al. (2020) [16]—with the lower bound of the 95% CI permanently exceeding 1.0. However, the 95% PI failed to establish predictive significance at any temporal point. Notably, leave-one-out sensitivity analysis (Figure 7B) revealed substantial structural instability driven by a single cohort; specifically, the omission of Fang et al. (2013), the LMR marker [1,2,5,13,16,17,21,24,27,28,29,51,52,55,56,57,58,61], and OS.

The omission of Fang et al. 2019 [9] shifted the 95% PI to strictly exclude the null value (1.31–1.86), whereas following the omission of any other study, the PI continued to cross 1.0. Consequently, further prospective studies are required to establish a stable and reproducible predictive association between NLR and DFS.

Cut-off thresholds ranged from 1.79 to 4.51, with a median of 2.42. Regarding the previous conclusion, no other analysis on cut-off thresholds was done.

#### 3.1.3. NLR and DSS

The primary meta-analysis for the association between NLR and DSS included 12 studies [2,5,12,14,16,21,31,32,36,53,57,62] that reported univariate results, of which eight also reported complete multivariate results. The pooled cohort comprised 2640 patients across six countries: three East Asian populations (China, Japan, and Taiwan) from six studies, and two other populations (Austria and the UK) from two studies.

Across the included studies (with results from multivariate analysis), the median sample size was 329 patients (range, 94–701), and the median follow-up period was 3.9 years (range, 1–7 years). The median of the median or mean patient age across seven of the eight cohorts was 60 years (range of medians, 39.9–69.6 years), and the median proportion of male patients per study was 64% (range, 42–93%). Early-stage disease (Stages I and II) was reported in four studies, which accounted for a median of 33% per study (range, 0–100%). The percentage of cases (in DSS) was reported or estimated in six studies, and the median was 17% (range, 12.2–37%).

**Figure 8:**
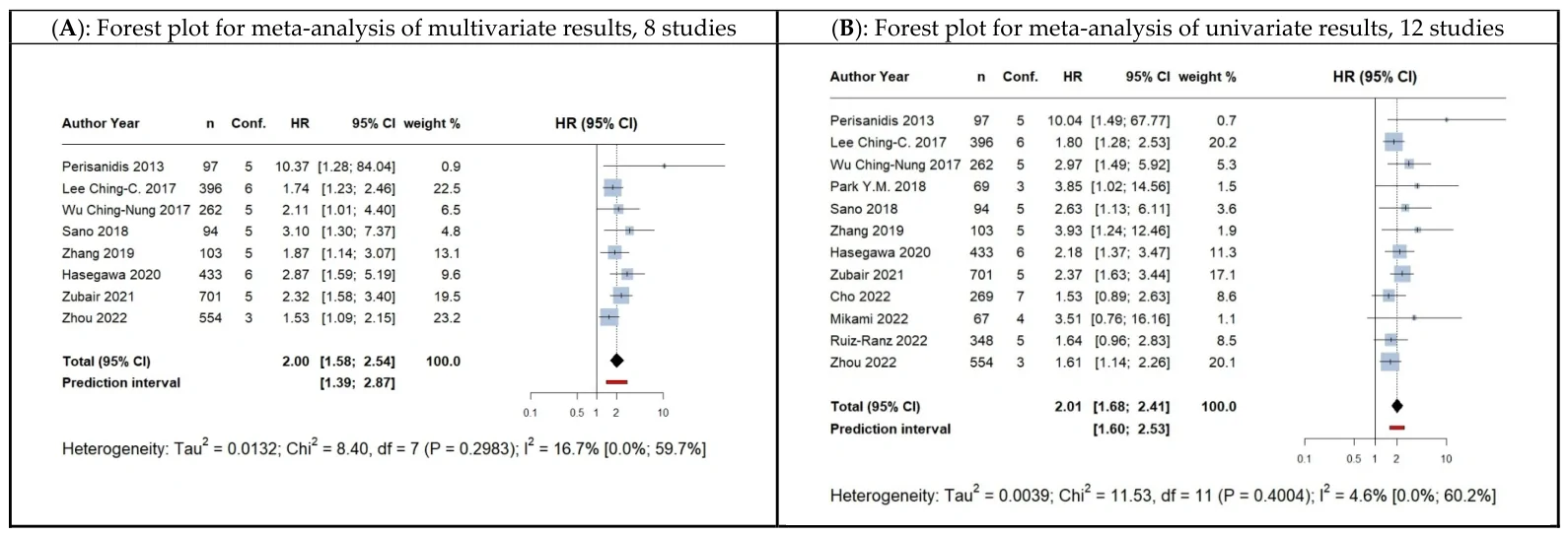
Forest plots for the meta-analysis of NLR and DSS: left, (**A**) primary meta-analysis of the multivariate results of 8 studies and, right, (**B**) secondary meta-analysis of the univariate results of 12 studies.

Tongue was the most frequently involved anatomical subsite and was reported in six studies, with a median percentage of 48% (range, 38–100%). Buc_Muc was reported in three studies, with a median percentage of 10% (range, 7–45%). Ging_Alveol was reported in two studies, with median percentages of 20% and 35%. Floor was reported in two studies, with a median percentage of 13% and 20%. Other_S was reported in two studies, with median percentages of 16% and 18%. Hard_P was reported in one study, with a median percentage of 0.4%. Retr_Tr and Lip were not reported in any of the studies. Regarding critical confounders, their median percentage included per study was 35.7% (range, 21–42.9%). A random-effects model was used for both primary and secondary meta-analyses, and the results are presented as forest plots in Figure 8, A and B, respectively.

**Figure 9:**
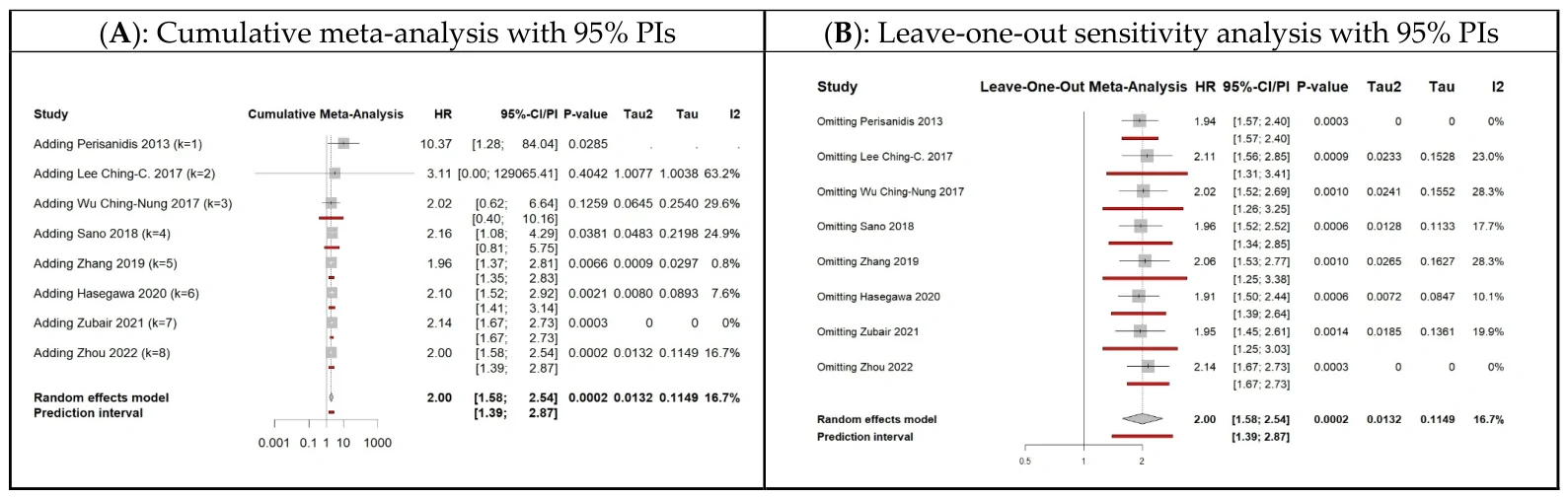
Forest plots: left, (**A**) cumulative meta-analysis of the multivariate data and, right, (**B**) leave-one-out sensitivity analysis for NLR and DSS. The plots also include 95% prediction intervals on the right of the red segments.

Notably, both meta-analyses exhibited non-significant, low to moderate between-study heterogeneity (secondary: I-squared = 4.6%, 95% CI: 0–60.2%, *p* = 0.400; primary: 16.7%, 95% CI: 0–59.7%, *p* = 0.298). In both models, the pooled hazard ratios, alongside their respective 95% confidence and prediction intervals, demonstrated a statistically significant association between elevated NLR and reduced DSS (Figure 8A,B). Specifically, utilizing the multivariate data in the primary analysis yielded a pooled HR of 2.00 (95% CI: 1.58–2.54, *p* = 0.0002; 95% PI: 1.39–2.87). Despite the limitation of a small sample of studies (k = 8), these statistics indicate that elevated preoperative NLR serves as a robust, independent prognostic factor for poor DSS. Furthermore, because the 95% PI strictly excludes the null value, this prognostic value is expected to remain significant across comparable future clinical populations. This conclusion is further corroborated by both the cumulative meta-analysis and the leave-one-out sensitivity analysis (Figure 9A,B).

Regarding cut-off thresholds ranging from 1.9 to 5.0, a non-significant negative linear correlation (r = -0.3, *p* = 0.4751) was observed between the log hazard ratios and the applied cut-off thresholds. However, a cumulative meta-analysis sorted by ascending cut-off values demonstrated that the lower bound of the 95% prediction interval (PI) permanently exceeded 1.0 at a threshold of 2.73. Consequently, the sample median (2.44) and the predictive stability threshold (2.73) represent empirically justified starting points for determining optimal cut-offs in future prospective trials.

### 3.2. PLR Marker

A total of 26 independent studies evaluated the prognostic value of the PLR marker. OS univariate results were reported in 19 studies and were estimated in four studies (23 studies in total), while multivariate results were given in 14 studies. For DFS, univariate results were reported and estimated in 10 and zero studies, respectively (10 studies in total), while multivariate results were given in five studies. Finally, for DSS, univariate results were reported and estimated in three and four studies, respectively (seven studies), while multivariate results were reported in four studies.

#### 3.2.1. PLR and OS

The primary meta-analysis of the association between PLR and overall survival (OS) included 14 of the 23 identified studies (reported complete multivariate results). The pooled cohort comprised 4088 patients across seven countries. Geographically, 11 studies evaluated East Asian populations (China, Japan, Republic of Korea, and Taiwan), while the remaining four evaluated other populations (Brazil, India, and Spain).

Across the included studies, the median sample size was 186.5 patients (range, 40–792), and the median follow-up period (reported in 13 studies) was 3.5 years (range, 2–5.5 years). The median of the median or mean patient age across the cohorts was 61.5 years (range of medians, 51.9–69.6 years), and the median proportion of male patients per study was 62% (range, 4.2–90.0%). Regarding tumor characteristics, early-stage disease (Stage I or II; reported in 12 studies) accounted for a median of 39.5% per study (range, 0–100%). The percentage of cases (deaths in OS) was reported or estimated in 11 studies, and the median was 26% (range, 8.3–69%).

Tongue was the most frequently involved anatomical subsite and was reported in 10 studies, with a median percentage of 38.5% (range, 17–100%). Buc_Muc was reported in eight studies, with a median percentage of 11% (range, 7–43%). Ging_Alveol was reported in six studies, with a median percentage of 19.5% (range, 11–35%). Floor was reported in seven studies, with a median percentage of 13% (range, 1–30%). Other_S was reported in four studies, with a median percentage of 23% (range, 10–36%). Hard_P was reported in three studies, with a median percentage of 4% (range, 2–15%). Retr_Tr was reported in four studies, with a median percentage of 10.5%% (range, 5–38%). Lip was reported in one study, with a percentage of 3%. The median percentage of critical confounders included per study was 36% (range, 21–71.4%). A random-effects model was used for the meta-analyses of both univariate and multivariate data.

**Figure 10:**
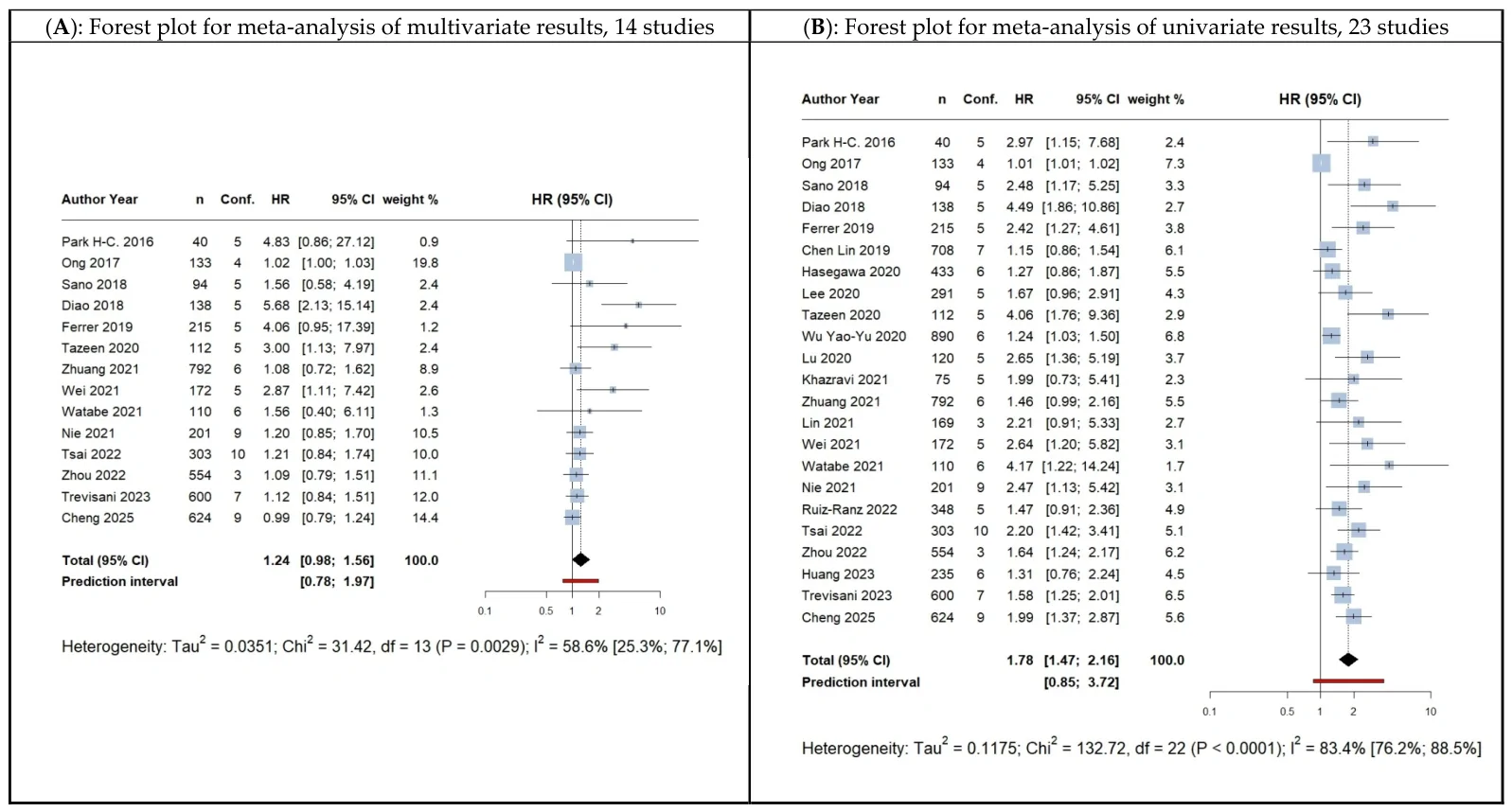
Forest plots for the meta-analysis of PLR and OS: left, (**A**) primary meta-analysis of the multivariate results of 14 studies and, right, (**B**) secondary meta-analysis of the univariate results of 23 studies.

Neither the primary nor the secondary meta-analysis demonstrated a statistically significant association between PLR and OS [2,3,5,14,16,17,19,21,25,26,27,28,29,35,51,52,54,55,56,58,59,60,61] (Figure 10). Specifically, both models exhibited moderate to severe between-study heterogeneity (primary: I-squared = 58.6%, 95% CI: 25.3–77.1%, *p* = 0.0029; secondary: I-squared = 83.4%, 95% CI: 76.2–88.5%, *p* < 0.0001). Furthermore, while the secondary analysis utilizing univariate data yielded a significant pooled HR (1.78, 95% CI: 1.47–2.16, *p* < 0.0001), its wide 95% prediction interval (0.85–3.72) crossed the null value, indicating severe prognostic instability across new cohorts. More critically, the primary analysis based on multivariate data (k = 14) failed to achieve statistical significance for either the pooled estimate (HR = 1.24, 95% CI: 0.98–1.56, *p* = 0.0655) or the prediction interval (95% PI: 0.78–1.97).

Subgroup analyses based on characteristics with no missing values (Table 3) failed to demonstrate statistically significant differences in pooled effect sizes across strata, a finding likely attributable to the limited statistical power inherent in the small subgroup sample sizes. However, exploratory evaluations revealed notable shifts in within-group heterogeneity when studies were dichotomized by publication year (before 2021, including the study by Zhuang et al. vs. at or after 2021 [25]; this threshold came from cumulative meta-analysis on the year of publication; Wei et al. 2021 was the first study with the lower bound of the 95% CI exceeding 1.0) [61], sample size (at most 186.5 vs. more than 186.5), proportion of male patients (at most 62% vs. more than 62%), and the number of adjusted critical confounders (at most five vs. more than five); the thresholds in the last three characteristics correspond to the medians. Furthermore, the applied PLR cut-off values exhibited extreme variability, ranging from 66.00 to 218.97 (median: 135.59). Consequently, although the current data are insufficient to support a definitive prognostic association between PLR and OS, it is worth mentioning that the results of the only large-scale prospective study by Zhuang et al. 2021 are against this association [25]; resolving this uncertainty will strictly require future prospective studies featuring more homogenized patient cohorts.

#### 3.2.2. PLR and DFS

**Figure 11:**
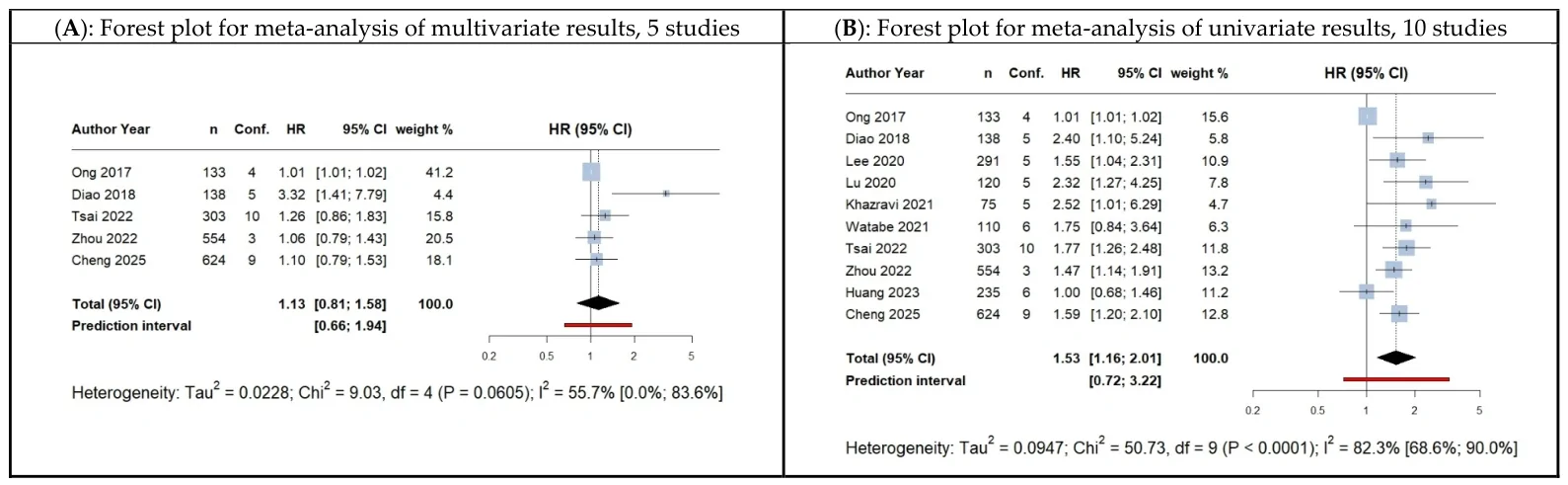
Forest plots for the meta-analysis of PLR and DFS: left, (**A**) primary meta-analysis of the multivariate results of 5 studies and, right, (**B**) secondary meta-analysis of the univariate results of 10 studies.

The meta-analysis evaluating the prognostic association between PLR and DFS is underpowered; thus, the results are summarized solely via forest plots (Figure 11). Univariate data were extracted from 10 studies, whereas multivariate data were available for only five. The primary pooled cohort comprised 1752 patients, drawn exclusively from China and Taiwan. Across these five primary studies, sample sizes ranged from 133 to 624 (median: 303), with male proportions varying widely between 4.2% and 90.0% (median: 53.0%). Study-level median ages spanned from 51.9 to 69.6 years, with median follow-up durations between 3.0 and 4.1 years. Furthermore, adjustments for critical confounders ranged from 21.0% to 71.4% (median: 36.0%). Both accompanying forest plots (Figure 11A,B) were generated utilizing random-effects models. Given these severe limitations, no definitive clinical conclusions can be drawn. Finally, the applied PLR cut-off thresholds demonstrated considerable variability, ranging from 110.7 to 170.2 (median: 129.0).

#### 3.2.3. PLR and DSS

**Figure 12:**
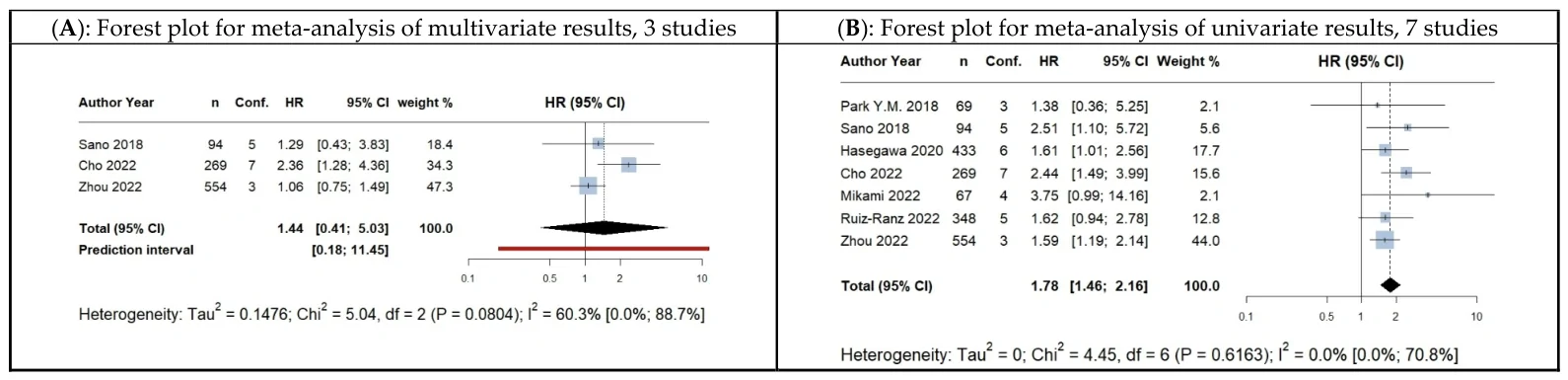
Forest plots for PLR and DSS: left, (**A**) primary meta-analysis of the multivariate results of 3 studies and, right, (**B**) secondary meta-analysis of the univariate results of 7 studies.

The association between PLR and DSS is presented by the two forest plots in Figure 12. Univariate data were analyzed with a fixed-effects model (I-squared = 0%, 95% CI: 0–70%, *p* = 0.6163), while for the analysis of multivariate data, a random-effects model was used. Univariate data were retained from seven studies, and multivariate data were retained from three studies. The pooled cohort of these three studies comprised 917 patients from China, Korea, and Japan, and more information, as well as the cut-off thresholds, can be extracted from Table 1.

### 3.3. LMR Marker

A total of 18 independent studies evaluated the prognostic value of the LMR marker [1,2,5,13,16,17,21,24,27,28,29,51,52,55,56,57,58,61]. OS univariate results were reported in 13 studies and were estimated in three studies (16 studies in total), while multivariate results were given in nine studies. For DFS, univariate results were reported and estimated in seven and zero studies, respectively (seven studies in total), while multivariate results were given in four studies. Finally, for DSS, univariate results were reported and estimated in one and three studies, respectively (four studies), while multivariate results were reported in none of these studies. To standardize the direction of effect across all included studies, hazard ratios (HRs) originally reported as ‘high vs. low’ were inverted (1/HR) to ‘low vs. high’ so that, as with NLR and PLR, effect sizes plotted to the right of the vertical null axis (HR > 1.0) consistently indicate that a low LMR is associated with reduced OS.

#### 3.3.1. LMR and OS

The primary meta-analysis for the association between LMR and OS included nine of the 16 identified studies (reported complete multivariate results). The pooled cohort comprised 2649 patients across four countries, three with East Asian populations (China, Japan and Malaysia) from eight studies and one study from Europe (Spain). The median sample size was 169 patients (range, 103–651), and the median follow-up period was 3 years (range, 2.6–5.5 years). The median of the median or mean patient age across the cohorts was 60 years (range of medians, 51.9–68 years), and the median proportion of male patients per study was 54% (range, 37–67.4%). Regarding tumor characteristics, early-stage disease (Stage I or II, reported in eight studies) accounted for a median of 56% per study (range, 25–100%). The percentage of cases (deaths in OS) was reported or estimated in 11 studies, and the median was 18.5% (range, 8.3–34%). Tongue was the most frequently involved anatomical subsite and was reported in seven studies, with a median percentage of 43% (range, 34–100%). Buc_Muc was reported in five studies, with a median percentage of 15% (range, 8–35%). Ging_Alveol was reported in three studies, with a median percentage of 17% (range, 10–26%). Floor was reported in three studies, with a median percentage of 6% (range, 5–19%). Other_S was reported in two studies, with percentages of 17% and 18%. Hard_P was reported in two studies, with percentages of 4% and 6%. Retr_Tr was reported in one study, with a median percentage of 38%. Lip was reported in three studies, with a median percentage of 2% (range, 2–4%). The median percentage of critical confounders included per study was 36% (range, 21–64%). Cut-off thresholds in eight of the nine studies that reported full multivariate results ranged from 2.60 to 5.0, and the median was 3.3. A random-effects model was used for the meta-analyses of both univariate and multivariate data.

**Figure 13:**
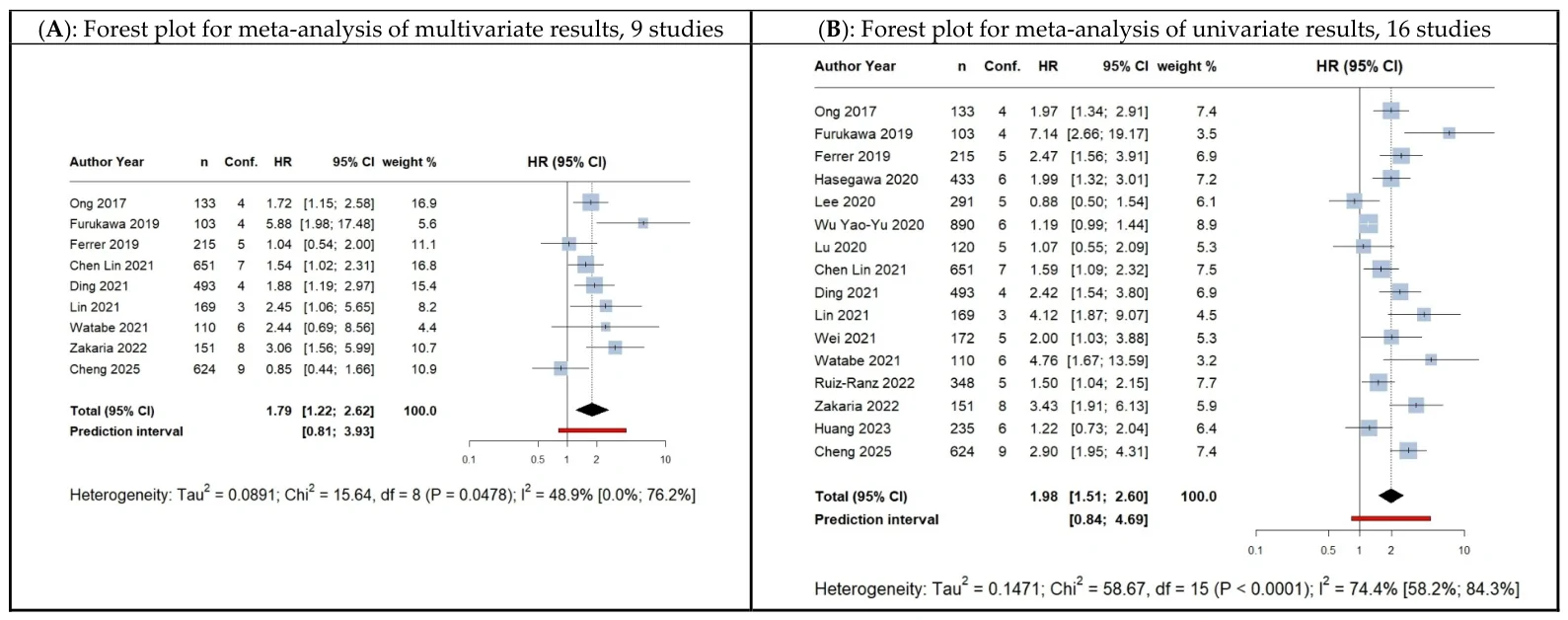
Forest plots for the meta-analysis of LMR and OS (note that after the inversion of HRs to ‘low vs. high’, point estimates positioned to the right of the vertical line of no effect demonstrate that low LMR is associated with poorer OS): left, (**A**) primary meta-analysis of the multivariate results of 9 studies and, right, (**B**) secondary meta-analysis on the univariate results of 16 studies.

**Figure 14:**
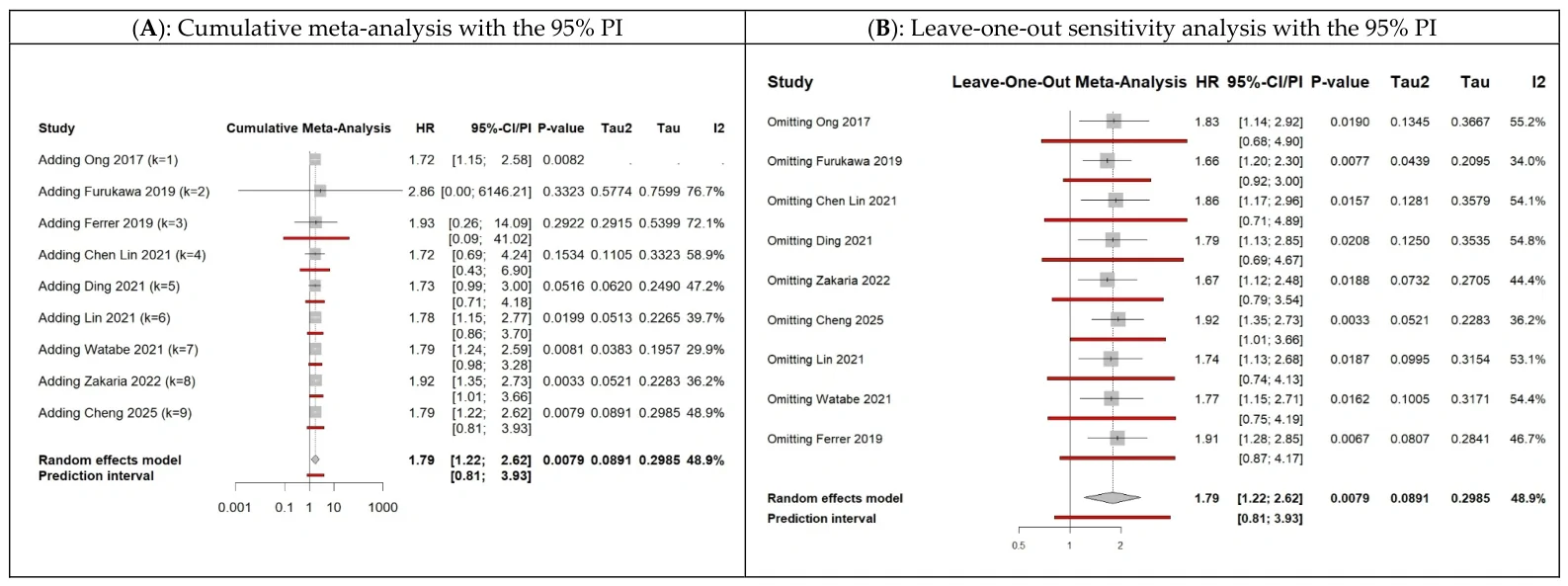
Forest plots: left, (**A**) cumulative meta-analysis of the multivariate data and, right, (**B**) leave-one-out sensitivity analysis for LMR and OS. The plots also include 95% prediction intervals on the right of the red segments.

A secondary meta-analysis of the univariate results of the 16 studies revealed severe heterogeneity, I-squared = 74.4%, 95% CI: 58.2–84.3%, *p* < 0.0001, while moderate heterogeneity was found in the analysis of the multivariate results given in nine studies, I-squared = 48.9%, 95% CI: 58.2–84.3%, *p* = 0.0478. In both analyses, statistically significant hazard ratios (transformed to low LMR vs. high LMR so that the results show the hazard regarding OS) were estimated; more precisely, in the primary meta-analysis, HR = 1.79, 95% CI: 1.22–2.62, and *p* = 0.0079, revealing that low LMR in the average may serve as an independent prognostic factor for poor OS. Unfortunately, in both analyses, the 95% PI strictly did not exclude the null 1.0 value (forest plots in Figure 13A,B). Regarding the primary meta-analysis, the 95% PI was found to range from 0.81 to 3.93; hence, the true prognostic effect of LMR on OS may not remain significant in future studies. The cumulative meta-analysis on the year of publication hazard ratios attained statistical significance after the study by Lin et al. 2021 [18] (the sixth study in the sequence), while the 95% PI excluded the null 1.0 value after the study by Zakaria et al. 2022 [13] (the seventh in the sequence); however, it became unstable again after the most recent study by Cheng et al. 2025 (Figure 14A) [52]. Further, the leave-one-out method of sensitivity analysis revealed, on the one hand, the stability of the HRs regarding the exclusion of any study, but, on the other hand, showed that the exclusion of just one study (Cheng et al. 2025) [52] leads to a significant improvement in the 95% PI (Figure 14B). Accordingly, regarding the limitation of the number of studies (k = 9 < 10), as well as the effect of the most recent study by Cheng 2025 [52], more studies are needed to confirm that low LMR is a significant independent prognostic factor of poor OS.

#### 3.3.2. LMR and DFS, DSS

**Figure 15:**
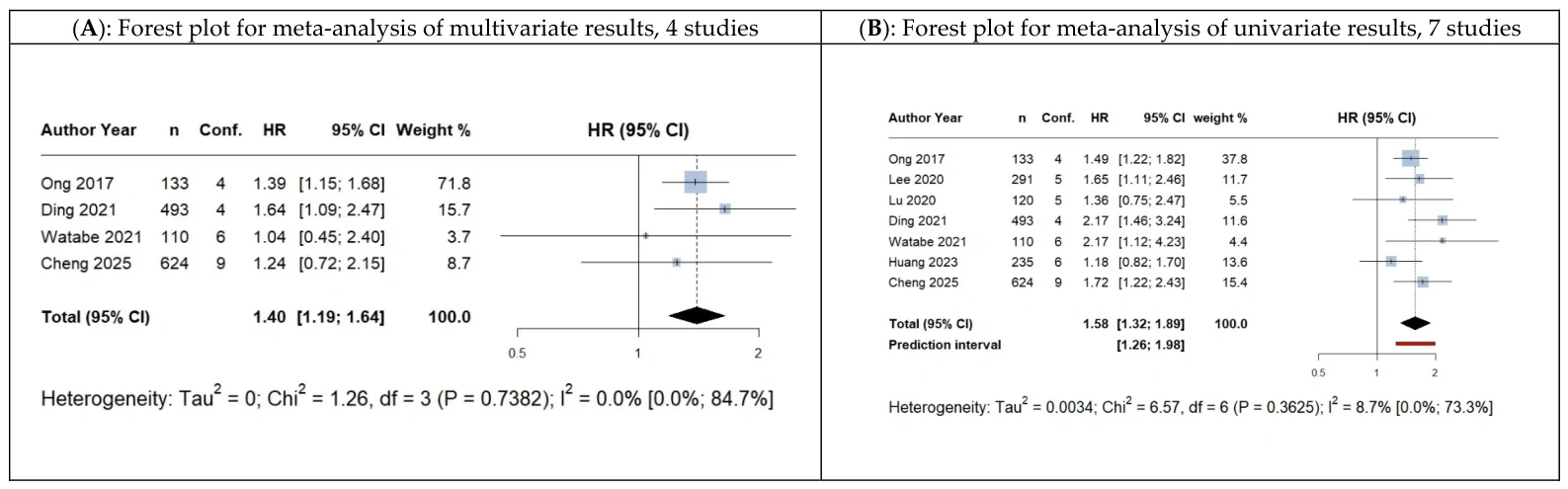
Forest plots for the meta-analysis of LMR and DFS (note that after the inversion of HRs to ‘low vs. high’, point estimates positioned to the right of the vertical line of no effect demonstrate that low LMR is associated with poorer DFS): left, (**A**) primary meta-analysis on the multivariate results of 4 studies and, right, (**B**) secondary meta-analysis on the univariate results of 7 studies.

**Figure 16:**
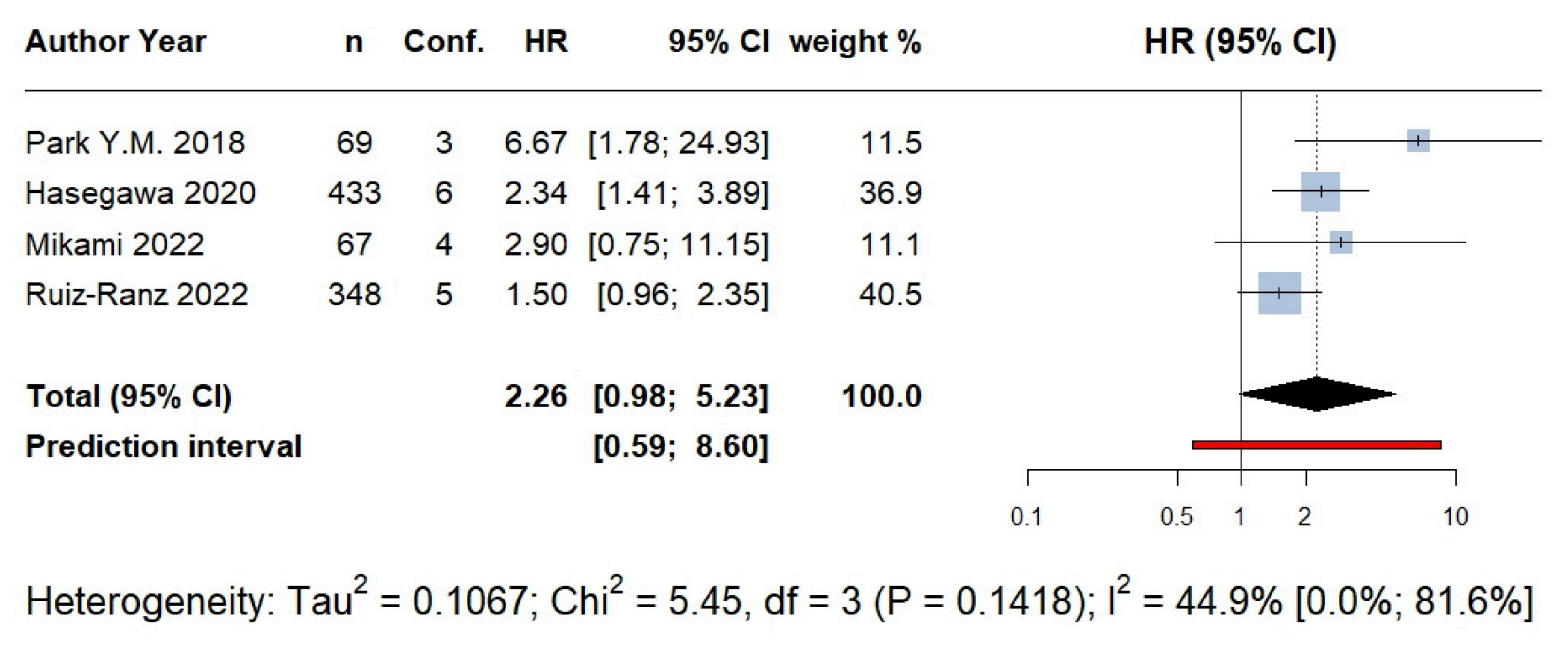
Forest plot for the meta-analysis of 4 studies that reported only univariate results for LMR and DSS (note that after the inversion of HRs to ‘low vs. high’, point estimates positioned to the right of the vertical line of no effect demonstrate that low LMR is associated with poorer DSS).

Regarding the association between LMR and DFS, in the literature, we found seven studies with univariate results, four of which reported full multivariate results (on 1360 patients from two East Asian countries, namely, China and Japan, with the percentage of males ranging from 52% to 56%). Forest plots are given in Figure 15. From an exploratory point of view, data from univariate results showed non-significant low heterogeneity, I-squared = 8.7%, 95% CI: 0–73.3%, *p* = 0.3625, and supported the hypothesis that low LMR is associated with poor DFS, HR = 1.58, 95% CI: 1.32–1.89, *p* = 0.0007, 95% PI: 1.26–1.98. On the other hand, the underpowered meta-analysis of the multivariate data revealed low heterogeneity, I-squared = 0%, 95% CI: 0–84.7%, *p* = 0.7382 (tau = 0), and the fixed-effects model, which could be used in this case, also supported the hypothesis that lower values of LMR are associated with poorer DFS, HR = 1.40, 95% CI: 1.19–1.64, *p* < 0.0001. Based on the above positive findings, more studies are needed to clarify the stability of the hypothesis that low LMR is a significant independent prognostic factor of poor DFS. Finally, regarding the association between preoperative LMR and DSS, four studies reported univariate results (the corresponding non-significant multivariate results were not reported), based on three retrospective studies from Japan, South Korea and Spain, from a total of 917 patients. Meta-analysis with a random-effects model revealed statistically non-significant moderate to severe heterogeneity, I-squared = 44.9%, 95% CI: 0–81.6%, *p* = 0.1418, and a non-significant correlation between preoperative low LMR and DSS: HR = 2.26, 95% CI: 0.98–5.23, 95% PI: 0.59–8.60 (forest plot is given in Figure 16). Consequently, the current data are insufficient to support any prognostic association between LMR and DSS.

## 4. Discussion

The results of this systematic review and meta-analysis suggest that preoperative peripheral blood inflammatory markers have potential prognostic relevance in oral squamous cell carcinoma (OSCC), although the strength and consistency of the evidence differ substantially across markers and endpoints [2,4,7,9,10,13,36,58]. Rather than supporting all markers equally, the present findings indicate that NLR shows the most consistent prognostic performance [1,2,12,14,16,19,21,25,26,27,31,32,51,52,53,57,60,62], whereas PLR and LMR require more cautious interpretation because statistical significance was not reproduced across all outcomes and the prediction intervals were not consistently robust [3,13,24,28,35,51,55,56,58]. These markers remain clinically attractive because they are inexpensive, non-invasive, and derived from routine blood tests [12,19,59], but their practical use should reflect the different levels of evidentiary support observed in this review [4,20,21,22].

Among the evaluated markers, NLR emerged as the most consistent prognostic indicator, particularly for OS and DSS. For OS, both the primary meta-analysis of multivariate data and the secondary meta-analysis of univariate data yielded statistically significant pooled hazard ratios. In both analyses, the 95% prediction intervals excluded 1.0, supporting the likelihood that the adverse prognostic effect of elevated preoperative NLR would persist in future comparable populations. This interpretation is further supported by the cumulative meta-analysis and sensitivity analyses, which suggest that elevated NLR may be considered a robust independent preoperative marker of poor overall survival in OSCC.

The association between elevated NLR and poor DSS was similarly strong. Both the primary and secondary analyses showed statistically significant pooled hazard ratios, low-to-moderate heterogeneity, and prediction intervals that remained above the null value, indicating that elevated preoperative NLR also functions as a robust independent prognostic factor for disease-specific survival (DSS). This is broadly in line with prior individual studies that identified NLR as an adverse prognostic marker in OSCC, especially for cancer-related mortality [10,12,31]. In addition, *Baixia Zhang* showed that younger OSCC patients generally had lower NLR values and better overall outcomes than a younger group while still demonstrating that NLR remained an independent predictor of DSS across age groups [31].

By contrast, the evidence for DFS should be presented more cautiously. Although the pooled hazard ratio showed a statistically significant association between elevated NLR and reduced DFS, the 95% prediction interval crossed 1.0, and the leave-one-out analysis showed that the apparent reproducibility of this finding was sensitive to the omission of Fang et al. (2013) [9], indicating instability and heterogeneity across studies. Therefore, it is more accurate to state that the current literature supports an association between elevated NLR and worse DFS, but it does not yet support a stable and reproducible prognostic effect across future cohorts [1,3,10,13,16,20,36,52,55,58].

LMR requires a more nuanced and cautious interpretation. The pooled analyses suggested that lower preoperative LMR was associated with poorer OS and DFS, which is biologically plausible given the balance between reduced lymphocyte-mediated anti-tumor immunity and increased monocyte-driven tumor-promoting activity [24,27,51]. The studies by Hui Shan Ong et al. and Sam Augustine Kandathil et al. also reinforce the relevance of LMR in OSCC, especially in early-stage disease [58].

However, for OS, both the primary and secondary analyses had prediction intervals that crossed 1.0, showing that this prognostic effect may not remain stable in future studies despite the statistically significant pooled hazard ratios. The doubt regarding the prognostic stability of LMR is further supported by Xu et al. [4]. Cumulative meta-analysis suggested that predictive significance became evident after the study by [56], but later evidence, including that of Cheng et al. (2025) [52], reintroduced uncertainty and made the prediction interval cross the null value again.

For DFS, the signal was more favorable, but it still requires caution because the primary multivariate evidence was clearly underpowered and based on a small number of studies [1,13,16,55,58]. Thus, it is more appropriate to state that low LMR is associated with worse DFS in the current literature while acknowledging that additional prospective studies are needed to confirm the stability and independence of this finding. For DSS, the presently available evidence does not support a statistically significant association between LMR and outcome, and the small number of available studies means this endpoint should be discussed as insufficiently supported rather than established (Hasegawa et al., 2020; Mikami et al., 2022; Y. M. Park et al., 2018) [2,21,58]. This shows that although LMR is biologically plausible and may have clinical relevance, its independent prognostic value still has to be validated in bigger and more homogeneous cohorts. Wu Yao-Yu et al. also proposed that LMR would even be superior to TNM staging in some cases, highlighting the possibility that immune-related biomarkers could capture biologic information that cannot be reflected by anatomical staging alone [4,27,51].

The prognostic role of PLR appears substantially less consistent than that of NLR [4,7]. In the primary multivariate meta-analysis for OS, elevated PLR was not significantly associated with poor overall survival; the prediction interval crossed the null value, heterogeneity was moderate to severe, and the only large prospective study cited in the dataset, Zhuang et al. (2021) [25], did not support a significant independent association. While some authors, such as Sanaz Tazeen et al. (2020) and Fa Chen et al. (2016), stated that PLR could be effective in predicting long-term survival and lymph node metastasis, our multivariate analysis for OS did not reach statistical significance (HR = 1.24, *p* = 0.0655) [20,51]. This discrepancy illustrates a major problem in the field of biomarker research: correlations found in univariate analyses often weaken once adjustments are made for tumor stage, comorbidity, or other inflammatory variables [7,25,53]. Accordingly, the safest interpretation is that the current literature does not support a statistically significant independent prognostic association between PLR and OS.

The same caution should be extended to DFS and DSS. For DFS, the evidence was underpowered, especially in the multivariate analysis, and the pooled estimate did not provide reliable support for an independent prognostic association; for DSS, only three multivariate studies were available, and the pooled estimate remained highly imprecise with a very wide prediction interval. PLR may still have exploratory value or correlate with adverse clinicopathologic features in some cohorts [20,51,54,63], but based on the present meta-analytic results, it should not be described as a robust stand-alone prognostic marker in OSCC [2,3,5,13,16,17,18,19,20,21,25,26,27,35,52,53,54,55,58,60,61]. The wide prediction interval (PI) for PLR (0.78–1.97) also indicates a large variability in effect estimates between studies. Importantly, the only large prospective cohort included in our review, that by Zhuang et al. (2021) [25] , did not indicate a significant independent connection between PLR and survival, which likely contributed to the decreased stability of the pooled estimate. On the other hand, some investigations reported that increased PLR is associated with adverse pathological features, including cervical lymph node involvement, and with worse DFS [53,54,63,64]. All things considered, PLR may still have exploratory value, but the current evidence does not support its use as a stand-alone prognostic marker [4].

In addition, the observed prognostic associations are biologically plausible in light of the interplay between systemic inflammation and tumor progression [4,5,65]. Neutrophils and platelets may promote tumor growth, angiogenesis, epithelial–mesenchymal transition, and immune evasion through cytokine- and growth factor-mediated signaling, whereas lymphocytes support anti-tumor immune surveillance and monocytes may contribute to tumor progression by differentiating into tumor-associated macrophages [3,5,16,18,19,27,66]. On this basis, inflammatory ratios provide a peripheral blood reflection of the balance between host immune defense and tumor-promoting inflammation [1,4,7].

From a clinical perspective, these findings suggest that inflammatory markers may complement TNM staging, but they should not be viewed as replacements for anatomical staging or established prognostic factors [7,16,18,32]. TNM remains essential for treatment planning, yet it does not capture biological heterogeneity or host immune status, which may help explain outcome differences among patients with the same stage [5,15,27,32]. In this context, NLR appears to be the most promising marker for risk stratification, whereas PLR and especially LMR require more cautious interpretation before routine clinical application [4,7].

Importantly, however, the independent prognostic association observed for NLR in the present meta-analysis should not be interpreted as evidence that NLR improves predictive performance beyond TNM staging. Although the primary analyses were based on multivariate hazard ratios and several included studies adjusted for TNM stage and other established clinicopathological factors, this demonstrates independent prognostic association rather than incremental predictive value. The present meta-analysis did not directly compare the discrimination, calibration, or clinical utility of TNM-based prognostic models with and without NLR. Some individual studies have reported that multivariable models or nomograms incorporating inflammatory indices alongside tumor stage and other clinicopathological variables may achieve higher concordance indices or improved time-dependent ROC performance compared with TNM staging alone [25,31]. However, these findings require independent prospective validation before it can be concluded that NLR provides clinically meaningful incremental predictive value beyond TNM staging. Future studies should therefore directly compare validated TNM-based models with and without NLR using appropriate measures of model performance and clinical utility.

Beyond TNM staging, a growing body of work has evaluated molecular biomarkers (e.g., p53, COX-2, PD-L1) and multi-gene prognostic signatures derived from transcriptomic or multi-omics data. These approaches can capture tumor-intrinsic biological heterogeneity and, in some cohorts, outperform purely anatomical staging (PMID: 42506409; PMID: 39842500). Their principal advantages are higher biological specificity and the potential for refined risk stratification. However, they generally require specialized tissue processing, sequencing or immunohistochemistry platforms, are more costly, and still lack standardized cut-offs and broad prospective validation, limiting routine clinical adoption. In contrast, systemic inflammatory indices such as NLR, PLR and LMR are derived from a routine preoperative complete blood count. They are inexpensive, rapidly available in virtually every clinical setting, and reflect the host–tumor immunological balance. Their main disadvantages are lower disease specificity (values can be altered by infection, corticosteroids, smoking or concurrent inflammatory conditions) and the current lack of universally accepted thresholds. Consequently, inflammatory ratios are best viewed as complementary, low-cost tools that can be readily integrated into multivariable nomograms alongside clinicopathological factors, rather than as replacements for molecular or gene signature models.

From a translational standpoint, several studies in oral squamous cell carcinoma have demonstrated that systemic inflammatory indices such as the NLR (and, in some series, PLR) retain independent prognostic value when assessed alongside conventional clinicopathological factors. In addition, composite models or nomograms incorporating these markers together with tumor stage and other baseline variables achieve higher concordance indices and superior time-dependent ROC performance compared with TNM staging alone [25,31].

Several biological and methodological issues remain unresolved. In principle, preoperative values of systemic inflammatory indices such as NLR, PLR and LMR could be influenced by non-oncologic factors (for instance, intercurrent infection, concomitant inflammatory conditions or the use of corticosteroids), which may add noise to their association with oncologic endpoints. Although some studies attempt to mitigate these effects through exclusion criteria, most available datasets do not systematically adjust for a broader range of potential confounders, underscoring the need for more carefully designed prospective studies to clarify the robustness of these markers. Moreover, factors such as smoking behavior and other chronic inflammatory comorbidities are rarely captured in detail in these prognostic models, and they may further influence baseline inflammatory indices; future prospective work should incorporate and adjust for these variables explicitly [4,22,31,63,67]. Second, the available evidence is heavily concentrated in East Asian cohorts, raising the possibility that regional or ethnic differences in baseline hematologic reference ranges, dietary habits and environmental exposures, or even tumor biology (e.g., differences in human papillomavirus-related versus tobacco/alcohol-related carcinogenesis), may modulate the observed hazard ratios, further underscoring the need for validation in more geographically and etiologically diverse cohorts before these markers can be adopted as universal prognostic tools [4,5,32].

All things together, these considerations reinforce the idea that while NLR, PLR, and LMR are unlikely to replace established anatomical and histopathological prognostic tools, they serve as accessible, cost-effective indicators of the host–tumor immunological balance that complements—rather than substitutes for—the TNM system [31,36,56]. Their eventual incorporation into everyday clinical decision-making in OSCC will likely depend on the development of standardized and prospectively validated cut-off values, incorporated within multivariable nomograms and tested across ethnically and clinically diverse patient populations, in order to ensure broad generalizability [4,25].

These biological and methodological considerations are directly relevant to personalized treatment decision-making in OSCC. Patients identified as higher risk based on elevated preoperative NLR may warrant closer postoperative surveillance and more intensive follow-up [3,4,12,20]. While these markers provide a valuable ‘low-cost lens’ into tumor biology, the available evidence emphasizes that these thresholds should not yet be used in isolation to guide treatment escalation [4,7,12].

Conversely, patients with low NLR and preserved (high) LMR—specifically the subgroup exhibiting high LMR combined with low PLR—may represent a more favorable-risk subgroup with significantly improved outcomes [1,58,66]. However, any implications for treatment de-escalation are still speculative because of the absence of prospective interventional evidence confirming the safety of such strategies in this patient group [4,58].

Ultimately, integrating these readily available hematologic ratios into individualized risk stratification pathways may contribute to more precise identification of patients who require intensified care [5,19]. Such an approach is increasingly being implemented in clinical research through the development of multivariable nomograms, which have demonstrated a superior ability to predict survival compared to traditional TNM staging. This is accomplished by capturing the crucial balance between host immune defense and tumor-promoting inflammation [3,25,27,56].

A major remaining barrier to implementation is the lack of standardized cut-off values, together with interstudy variability in populations, analytic methods, and regional representation. Although cumulative analyses suggested potentially informative thresholds for NLR, these should be considered hypothesis-generating rather than directly transferable to all settings. The predominance of East Asian cohorts further limits generalizability, underscoring the need for prospective multicenter validation with harmonized thresholds and broader geographic coverage cut-off values and methodologies [4,7].

As previously mentioned, a major limitation to clinical implementation is the lack of established cut-off values. In this review, NLR cut-offs varied widely from 1.31 to 5.0, reflecting heterogeneity in study populations, analytic methods, and institutional practices. Cumulative meta-analysis, however, identified values associated with predictive stability, specifically approximately 2.90 for OS and 2.73 for DSS. Such values may serve as useful starting points for future prospective validation investigations.

Subgroup analyses also indicated that the relationship between NLR and OS could be stronger in patients over 60 years of age, which could be indicative of age-related inflammatory alterations. Furthermore, the prognostic effect appeared more consistent in East Asian cohorts than in non-Asian populations, although this interpretation is limited by the small number of research studies from other geographic regions. These findings underscore the need for caution when generalizing cut-off thresholds across populations.

### Strengths and Limitations

A major strength of this review is its comprehensive evaluation of three readily available inflammatory biomarkers—NLR, PLR, and LMR—within a single prognostic framework for OSCC. The analysis incorporates a substantial amount of evidence and is primarily focused on adjusted multivariate estimates, thus minimizing confounding bias. Moreover, the use of prediction intervals, cumulative meta-analysis, leave-one-out sensitivity analyses, and publication bias adjustment increases the methodological rigor and boosts the reliability of the findings. Also, the study is of high clinical relevance, since these markers are inexpensive, routinely available, and easily applicable in everyday practice [12,19,59]. Finally, the discussion is supported by a biologically plausible rationale linking systemic inflammation with tumor development and survival, which reinforces the translational relevance of the findings.

There are, nonetheless, several limitations that should be taken into consideration. Most of the included studies were retrospective, thus increasing the risk of selection bias and residual confounding. First and foremost is the strong geographic and ethnic concentration of the included evidence. The large majority of studies were retrospective and conducted in East Asian populations (approximately 80% of patients from China, Taiwan, Japan and South Korea). Baseline hematological reference ranges, dietary habits, prevalence of chronic inflammatory comorbidities, and the relative contribution of tobacco/alcohol versus other etiological factors (including HPV-related carcinogenesis) may differ substantially across ethnic and geographic groups. As a result, both the absolute cut-off values and the magnitude of the observed hazard ratios may not be directly generalizable to Western, South Asian, African or Latin American populations. Subgroup analyses already suggested attenuated or non-significant associations in the few non-East Asian cohorts, although the small number of such studies limits statistical power. Prospective multicenter validation in ethnically and geographically diverse populations, using harmonized laboratory methods and pre-specified thresholds, is therefore required before these markers can be recommended for routine use outside East Asian settings.

Additionally, other comorbidities and concurrent conditions (e.g., infections, autoimmune disorders, chronic inflammatory diseases, or medications affecting hematopoiesis) can alter peripheral blood cell counts and thereby influence NLR, PLR, and LMR values. Most of the included studies did not systematically exclude or adjust for these factors, so residual confounding from non-cancer-related inflammatory states remains possible despite the use of multivariate models.

Moreover, some primary studies reported the lip as an anatomical subsite without clearly distinguishing between the wet mucosal and dry lip, introducing a potential source of anatomical heterogeneity.

Publication bias was also evident, suggesting possible underrepresentation of small negative studies. In addition, the lack of standardized cut-off values and the prevalence of East Asian cohorts limit the generalizability of the findings [16,27,51,56]. We emphasize the critical need for large-scale, prospective validation in Western and diverse ethnic cohorts to evaluate potential differences in baseline inflammatory responses. Finally, the evidence for some endpoints, namely, PLR and DSS, is still restricted to relatively few research studies. These biomarkers and their clinical and therapeutic implementation should be validated in future prospective multicenter studies with consistent thresholds and with larger geographic representation.

Finally, a further limitation is that Embase and Web of Science were not included in the search. Our primary search strategy combined PubMed/MEDLINE, Scopus, BASE, Google Scholar and ScienceDirect, supplemented by manual screening of reference lists. Institutional access to Embase and the full Web of Science Core Collection was limited at the time of the searches. Given the substantial bibliographic overlap already captured by PubMed, Scopus and BASE, the additional yield expected from Embase/Web of Science was judged to be likely modest. To further reduce the risk of missing eligible studies, we performed extensive backward citation searching of all included articles and related systematic reviews. We acknowledge that the omission of Embase and Web of Science remains a potential limitation.

## 5. Conclusions

Preoperative inflammatory markers show heterogeneous prognostic performance in OSCC, with NLR providing the most consistent evidence, particularly for overall survival and disease-specific survival. In contrast, the current evidence does not support statistically significant independent prognostic associations for PLR across the examined endpoints, while LMR shows promising but still unstable associations for OS and DFS and no convincing evidence for DSS. These findings support further prospective multicenter validation before broad clinical implementation, especially for cut-off standardization and for confirmation in geographically diverse populations.

## Figures and Tables

**Table 1:** Characteristics of the 39 included studies. Cases follow the order of endpoints for each value.

Author	Year of Publication	Country	Area	Total N	Cases %	Age (Mean or Median)	Follow-Up	Start Year	End Year	Biomarkers (Cut-Off)	Endpoints	Confounders (Total Number Included)
Fang	2013	Taiwan	East Asian	226	17, 17	52.0 (27–84)	5 years (follow-up until July 2012 (median NR))	2007	2012	NLR (2.44)	DFS, OS	TNM staging, nodal status with ECS, histological differentiation (3)
Perisanidis	2013	Austria	Other	97	18	58.0	3.7 years (median 3.7 years (0.3–9.5))	2001	2009	NLR (1.9)	DSS	Age, smoking status, clinical TNM staging, histological differentiation, PNI (5)
Nakashima	2016	Japan	East Asian	124	23, 34	67.2	5 years	2003	2009	NLR (2.4)	OS, DFS	Age, cancer subtype, pTNM staging, histological differentiation (4)
Park H-C	2016	Republic of Korea	East Asian	40		66	3 years (35.58 months)	2004	2011	NLR (1.88), PLR (124.8)	OS	Age, pTNM staging, histological, PNI, cancer subsite (5)
Bobdey	2017	India	Other	471	51	50.0 (25–85)	2 years (median 22 months (0–98))	2007	2008	NLR (2.38)	OS	TNM staging, age, smoking status, alcohol consumption (4)
Lee Ching-Chih	2017	Taiwan	East Asian	396	36	53.0	2.4 years (median 2.4 years (range 1–9))	2009	2013	NLR (2.73)	DSS	Age, TNM staging, pTNM (advanced pT status—T3,T4, advanced pN status—pN2), PNI, histological differentiation, cancer subsite (Tongue–Buccal area), marginal status (6)
Ong	2017	China	East Asian	133	8.3, 8.3	52.0 (24–74)	5 years (median 52 mo (7–72); min 36 mo for survivors)	2009	2013	LMR (3.2), NLR (NR), PLR (129.0)	DFS, OS	Age, pTNM, histological differentiation, PNI (4)
Wu Ching-Nung	2017	Taiwan	East Asian	262	17.2, 12.2, 21.8	51.0 (24–85)	6 years (mean 67.1 months (2–137))	2004	2011	NLR (2.95)	DFS, DSS, OS	Age, smoking, alcohol consumption, pTNM staging, ENE (5)
Kao	2018	Taiwan	East Asian	613	32	53.0	3 years (follow-up until Dec 2016 (median NR))	2005	2014	NLR (2.28)	OS	Age, smoking, alcohol consumption, pTNM staging, ENE, histological differentiation, surgical margins, cancer subsite (8)
Park	2018	Republic of Korea	East Asian	69	21.1	62.0	4 years (mean 50.6 months (3–117))	2007	2016	LMR (2.91), NLR (2.29), PLR (131.0)	DSS	Age, cancer subsite, TNM classification–TNM staging (3)
Sano	2018	Japan	East Asian	94		67 (20–94)	3 years (median 41.6 months (3.0–107.6))	2007	2015	NLR (2.36), PLR (138.47)	OS	Age, smoking status, cTNM, cancer subsite, postoperative radiotherapy/chemoradiotherapy (5)
Diao	2018	China	East Asian	138	15, 22	60	4 years (median 48 months) (4–134)	2006	2016	NLR (2.9), PLR (170.2)	OS, DFS	Age, smoking, alcohol history, pTNM staging, histological differentiation (5)
Furukawa	2019	Japan	East Asian	103	15.1	63.0	5 years (5-year follow-up (capped); median NR)	2001	2015	LMR (4.29)	OS	Age, smoking status, alcohol consumption, cTNM (4)
Zhang	2019	China	East Asian	103	15	patients ≤ 40 matched with patients ≥ 60	7 years (mean 89.9 months (range 7–205))	2008	2012	NLR (2.56)	DSS	Smoking status, alcohol consumption, TNM staging, PNI, cancer subsite, adjuvant therapy (5)
Chen Lin	2019	China	East Asian	708	60	60	3.6 years (median 42.9 months)	2002	2016	NLR (2.03), PLR (139.77)	OS	Age, pTNM staging, smoking, alcohol consumption, histological differentiation, cancer subtype, adjuvant therapy (7)
Ferrer	2019	Spain	Other	215	34	67.5	5 years (median 41 months) (7–87)	2011	2014	NLR (3), PLR (66), LMR (2.6)	OS	Age, pTNM staging, cancer subtype, histological differentiation, surgical margins (5)
Hasegawa	2020	Japan	East Asian	433	16, 23	66.0 (22–92)	5 years (mean 59.1 months)	2001	2013	LMR (4.35), NLR (2.22), PLR (134.3)	DSS, OS	Age, smoking status, alcohol consumption, TNM classification, histological differentiation, extranodal extension (pENE+) (6)
Lee	2020	Republic of Korea	East Asian	291	23, 17.2	63.0 (24–91)	4 years (mean 41 months (range 3–144))	2005	2018	LMR (4.45), LMR (4.65), NLR (2.16), NLR (2.23), PLR(131.07), PLR (135.14)	DFS, OS	Age, pTNM, PNI, surgical marginal status, adjuvant therapy (5)
Tazeen	2020	India	Other	130	22	51.5 (16–80)	2 years (6–29 months)	2016	2018	NLR (3.1), PLR (142.0)	OS, DFS	Age, cancer subsite, TNM staging, histological differentiation, adjuvant therapy (5)
Wu Yao-Yu	2020	Taiwan	East Asian	890		50.8 (44–57)	6 years (median 72.7 months (survivors; IQR 14.9–101.4))	2005	2012	LMR (4.21), NLR (2.9), PLR (110.6)	OS	Age, smoking status, alcohol consumption, TNM staging, histological differentiation, adjuvant therapy (6)
Lu	2020	China	East Asian	120	39, 31	55 (22–86)	3.1 years	2012	2017	NLR (2.8), LMR (4.02), PLR (140.5)	OS, DFS	Age, DOI, pTNM staging, histological differentiation, adjuvant therapy radiotherapy/chemotherapy/chemoradiotherapy (5)
Chen Lin	2021	China	East Asian	651	20	60.0	3 years (median 31.1 mo (IQR 17.7–49.8))	2010	2017	LMR (3.18)	OS	Age, smoking status, alcohol consumption, histological type, TNM staging, tumor differentiation, adjuvant therapy (7)
Ding	2021	China	East Asian	493	6	60.0	3 years (follow-up until 31 August 2018 (median NR))	2012	2015	LMR (3.4), NLR (2.9)	DFS, OS	Age, smoking, TNM staging, cancer subsite (4)
Khazravi	2021	Iran	Other	129	35	58.0 (40–72)	5 years (up to 96 mo; mean OS 65.8 mo; survival set n = 75)	2013	2018	NLR (1.21), PLR (97.81)	DFS, OS	Age, smoking, alcohol consumption, TNM staging, PNI (5)
Zhuang	2021	China	East Asian	792	26	60.0 (51–68)	2 years (median follow-up of survivors 27.48 months)	2002	2018	NLR (1.31), PLR (218.97)	OS	Age, smoking, alcohol consumption, TNM staging, histological differentiation, adjuvant therapy (6)
Zubair	2021	UK	Glasgow	701			1 years (12 months)	2006	2019	NLR (5)	DSS	PNI, DOI, pTNM staging, histological differentiation, ENE (+) (5)
Lin	2021	China	East Asian	169	21	57	4.2 years (median 50 months (1–130))	2008	2019	NLR (1.61), PLR (177.86), LMR (4.15)	OS	Age, pTNM staging, adjuvant therapy radiotherapy/chemotherapy/chemoradiotherapy (3)
Wei	2021	China	East Asian	172		69 (25–88)		2008	2019	NLR (2.85), PLR (177.4), LMR (3.4)	OS	Age, smoking, alcohol consumption, pTNM staging, PNI (5)
Watabe	2021	Japan	East Asian	110	18	68	5.5 years	2004	2012	NLR (1.79), PLR (114.97), LMR (5)	OS, DFS	Age, pTNM staging, histological differentiation, PNI, ENE (+), surgical margins (6)
Nie	2021	China	East Asian	201	44	62	4.5 years (2–95 months)	2007	2020	NLR (2.8), PLR (162.5)	OS	Age, smoking, PNI, pTNM staging, histological differentiation, cancer subtype, DOI, ENE (+), surgical margins (9)
Cho	2022	Korea	East Asian	269	24	55.1 (18–90)	3 years (36 months (range 0–185))	2013	2019	NLR (1.75) PLR (159.45)	DSS	Age, cancer subsite, TNM staging, histological differentiation, DOI, PNI, adjuvant therapy (7)
Mikami	2022	Japan	East Asian	72	13	68.2 (26–92)	6 years (mean 71.8 months)	2016	2012	LMR (6.41), NLR (1.47), PLR (135.3)	DSS	Age, cancer subsite, TNM classification, histological differentiation (4)
Ruiz-Ranz	2022	Spain	Other	348	35, 46	63.0 (28–92)	5 years (median 54 months)	1996	2007	LMR (4.58), NLR (4.08), PLR (205.0)	DSS, OS	Age, smoking, alcohol consumption, cancer subsite, TNM staging, (5)
Tsai	2022	Taiwan	East Asian	303	48, 30	57.0 (31–86)	4 years (median 40.9 months (range 1.4–122.7))	2008	2017	NLR (4.51), PLR (119.34)	DFS, OS	Age, smoking, alcohol consumption, TNM staging, cancer subsite, depth of invasion (DOI), ENE, surgical margins (5 mm), histological grade, PNI (10)
Zakaria	2022	Malaysia	East Asian	151		59.7	3 years (median 30 months (range 1–217))	2000	2020	LMR (NR)	OS, DFS	Age, smoking, alcohol consumption, histological differentiation, TNM staging, cancer subtype, histological differentiation, adjuvant therapy (8)
Zhou	2022	China	East Asian	554	37, 37, 37	69.6	4.1 years (median 49 months), (1–76)	2010	2012	NLR (2.18), PLR (110.7)	OS, DFS, DSS	Age, pTNM staging, histological differentiation (3)
Huang	2023	Taiwan	East Asian	235	56, 68	71.0 (67–75)	3.5 years (median 42 months (6–186))	2011	2020	LMR (4.0), NLR (2.9), PLR (135.6)	DFS, OS	Age, smoking, alcohol consumption, cancer subtype, pTNM staging, adjuvant therapy (6)
Trevisani	2023	Brazil	Other	600	49	61.3 (15–91)	3 years (mean 33.1 months (0–133))	2009	2018	NLR (3.38), PLR (167.3)	OS	Age, cancer subtype, PNI, pTNM staging, histological differentiation, ENE (+), surgical margins (7)
Cheng	2025	China	East Asian	624	19, 23	60	3 years (median 35 months)	2016	2021	NLR (2.3), LMR (3), PLR (132.7)	OS, DFS	Age, DOI, pTNM staging, histological differentiation, PNI, ENE (+), smoking history, alcohol consumption, cancer subtype (9)

NLR = neutrophil-to-lymphocyte ratio; PLR = platelet-to-lymphocyte ratio; LMR = lymphocyte-to-monocyte ratio; NR = not reported. Area refers to geographic/ethnic classification. Cut-off values shown in parentheses after biomarker name. The number in parentheses after multivariate factors (last column in table) indicates how many of these factors are from the pre-specified core set of 14 important prognostic factors (pre-treatment clinical factors: 1. clinical tumor classification (cTNM)- Clinical TNM staging, 2. radiologic extranodal extension (rENE+), and 3. cancer subsite; post-treatment pathological factors: 4. pathological tumor classification (pTNM)—pathological TNM staging, 5. extranodal extension ENE (+)-ECS, 6. depth of invasion (DOI), 7. perineural invasion (PNI), 8. surgical margin status (involved margins), 9. degree of differentiation, 10. histological type, and 11. adjuvant therapy radiotherapy/chemotherapy/chemoradiotherapy; behavioral factors: 12. Smoking history, 13. alcohol consumption, and 14. age).

**Table 2:** Results of subgroup analyses; *k* is the number of studies in each group.

Subgroup (k)	HR	95% CI	*p*-Value Subgroup	*I* ^2^	95% CI	*p*-Value (Test)
Year						
<2021 (12)	1.48	1.24–1.77	<0.001	48.2%	0.0–73.4%	0.702
≥2021 (8)	1.57	1.19–2.07	0.007	0.0%	0.0–67.6%	
Area						
East Asian (16)	1.54	1.31–1.81	<0.001	48.5%	8.1–71.1%	0.637
Other (4)	1.42	0.85–2.36	0.117	0.0%	0.0–84.7%	
Follow-up						
≤4 (12)	1.57	1.28–1.92	<0.001	5.3%	0.0–60.5%	0.515
>4 (8)	1.44	1.16–1.79	0.005	51.5%	0.0–78.3%	
Sample size						
≤276.5 (10)	1.54	1.19–1.98	0.004	41.2%	0.0–71.9%	0.916
>276.5 (10)	1.51	1.25–1.82	<0.001	26.7%	0.0–64.7%	
Males (%)						
≤64% (11)	1.45	1.19–1.75	0.002	35.3%	0.0–68.2%	0.395
>64% (9)	1.62	1.28–2.04	0.001	34.7%	0.0–69.9%	
Confounders						
≤5 (11)	1.53	1.25–1.87	<0.001	41.0%	0.0–70.9%	0.960
>5 (9)	1.52	1.20–1.93	0.004	34.3%	0.0–69.8%	
Age						
≤60 (11)	1.39	1.17–1.64	0.001	43.9%	0.0–72.2%	0.093
>60 (9)	1.73	1.35–2.23	0.001	0.0%	0.0–64.8%	

**Table 3:** Results of subgroup analyses; *k* is the number of studies in each group.

Subgroup (k)	HR	95% CI	*p*-Value	I-Squared	95% CI	p-Subgroup
Year						
<2021 or Zhuang (7)	1.54	0.83–2.84	0.137	75.0%	46.8–88.2%	0.364
≥2021 (7)	1.19	0.86–1.64	0.234	0.0%	0.0–70.8%	
Area						
East Asian (11)	1.21	0.93–1.57	0.130	56.1%	13.8–77.7%	0.597
Other (3)	1.46	0.35–6.03	0.371	67.0%	0.0–90.5%	
Sample size						
≤186.5 (7)	1.77	0.95–3.30	0.066	76.4%	50.3–88.7%	0.131
>186.5 (7)	1.15	0.84–1.58	0.322	0.0%	0.0–70.8%	
Males (%)						
≤62% (7)	1.27	0.79–2.04	0.270	72.4%	40.5–87.2%	0.964
>62% (7)	1.25	0.91–1.72	0.133	0.0%	0.0–70.8%	
Confounders						
≤5 (8)	1.60	0.93–2.74	0.079	75.6%	51.0–87.8%	0.188
>5 (6)	1.13	0.79–1.60	0.421	0.0%	0.0–74.6%	
Age						
≤61.5 (7)	1.17	0.82–1.68	0.321	66.6%	25.6–85.1%	0.404
>61.5 (7)	1.41	0.94–2.12	0.083	31.4%	0.0–70.8%	

## Data Availability

The original contributions presented in this study are included in this article. Further inquiries can be directed to the corresponding author.

## Supplementary Materials

The following supporting information can be downloaded at https://www.mdpi.com/article/10.3390/ijms27177638/s1.
