# Supplementary material for: Preoperative Inflammatory Blood Indices as Prognostic Markers in Oral Squamous Cell Carcinoma: A Systematic Review and Meta-Analysis
Source: Int J Mol Sci. 2026 Aug 26;27(17):7638. doi: 10.3390/ijms27177638 (PMC13566896; doi:10.3390/ijms27177638)
Supplement: Supplementary file 1 [file ijms-27-07638-s001.zip › ijms-4483454-supplementary.pdf]

Table S1- search string

Database	Search string
PubMed /Medline - 644 <from inception to April 30 2026>	("Carcinoma, Squamous Cell"[Mesh] OR "Mouth Neoplasms"[Mesh] OR "Tongue Neoplasms"[Mesh] OR "oral squamous cell carcinoma"[tiab] OR OSCC[tiab] OR "oral cancer"[tiab] OR "oral cavity cancer"[tiab] OR "oral cavity carcinoma"[tiab] OR "mouth cancer"[tiab] OR "mouth neoplasm*"[tiab] OR "tongue cancer"[tiab] OR "tongue squamous cell carcinoma"[tiab]) AND ("neutrophil to lymphocyte ratio"[tiab] OR "neutrophil-to-lymphocyte ratio"[tiab] OR NLR[tiab] OR "platelet to lymphocyte ratio"[tiab] OR "platelet-to-lymphocyte ratio"[tiab] OR PLR[tiab] OR "lymphocyte to monocyte ratio"[tiab] OR "lymphocyte-to-monocyte ratio"[tiab] OR LMR[tiab] OR "inflammatory biomarker*"[tiab] OR "inflammatory marker*"[tiab] OR "hematologic marker*"[tiab] OR "haematologic marker*"[tiab] OR "hematologic parameter*"[tiab] OR "systemic inflammatory marker*"[tiab]) AND ("Prognosis"[Mesh] OR "Survival Analysis"[Mesh] OR "Disease-Free Survival"[Mesh] OR prognos*[tiab] OR survival[tiab] OR "overall survival"[tiab] OR "disease-free survival"[tiab] OR "disease specific survival"[tiab] OR "cancer specific survival"[tiab] OR outcome*[tiab] OR recurrence[tiab] OR recurren*[tiab])
Scopus - 228 <from inception to April 30 2026>	TITLE-ABS-KEY (("oral squamous cell carcinoma" OR OSCC OR "oral cancer" OR "oral cavity cancer" OR "oral cavity carcinoma" OR "mouth cancer" OR "mouth neoplasm*" OR "tongue cancer" OR "tongue squamous cell carcinoma") AND ("neutrophil to lymphocyte ratio" OR "neutrophil-to-lymphocyte ratio" OR NLR OR "platelet to lymphocyte ratio" OR "platelet-to-lymphocyte ratio" OR PLR OR "lymphocyte to monocyte ratio" OR "lymphocyte-to-monocyte ratio" OR LMR OR "inflammatory biomarker*" OR "inflammatory marker*" OR "hematologic marker*" OR "haematologic marker*" OR "hematologic parameter*" OR "systemic inflammatory marker*") AND (prognos* OR survival OR "overall survival" OR "disease-free survival" OR "disease specific survival" OR "cancer specific survival" OR outcome* OR recurrence OR recurren*)) AND (LIMIT-TO(LANGUAGE, "English"))
ScienceDirect- 100 <from inception to April 30 2026>	(OSCC OR "oral squamous cell carcinoma") AND (preoperative OR pretreatment) AND (NLR OR PLR OR LMR) AND (survival OR prognosis)
Google Scholar- 141 <from inception to April 30 2026>	"oral squamous cell carcinoma" NLR survival; "oral squamous cell carcinoma" PLR prognosis; "oral squamous cell carcinoma" LMR survival; "oral cancer" "neutrophil to lymphocyte ratio" outcome; "oral cancer" "platelet to lymphocyte ratio" survival; "tongue cancer" LMR prognosis
Base- 294 <from inception to April 30 2026>	("oral squamous cell carcinoma" OR OSCC OR "oral cancer" OR "tongue cancer")<br>AND<br>(NLR OR PLR OR LMR OR "neutrophil-to-lymphocyte ratio" OR "platelet-to-lymphocyte ratio" OR "lymphocyte-to-monocyte ratio")<br>AND<br>(prognos* OR survival OR "overall survival" OR "disease-free survival")

Table S2

PRISMA 2020 for Abstracts Checklist

Section and Topic	Item #	Checklist item	Reported (Yes/No)
**TITLE**			
Title	1	Identify the report as a systematic review.	Yes – Explicitly identified as a systematic review and meta-analysis
**BACKGROUND**			
Objectives	2	Provide an explicit statement of the main objective(s) or question(s) the review addresses.	Yes – Explicit aim to evaluate preoperative NLR, PLR and LMR as prognostic markers in OSCC
**METHODS**			
Eligibility criteria	3	Specify the inclusion and exclusion criteria for the review.	Yes – Studies reporting preoperative ratios and survival outcomes (OS, DSS or DFS) in surgically treated OSCC
Information sources	4	Specify the information sources (e.g. databases, registers) used to identify studies and the date when each was last searched.	Yes – PubMed/MEDLINE, Scopus, Base, Google Scholar and ScienceDirect from inception to 30 April 2026
Risk of bias	5	Specify the methods used to assess risk of bias in the included studies.	Yes – QUIPS tool
Synthesis of results	6	Specify the methods used to present and synthesise results.	Yes – Random-effects meta-analyses of multivariate HRs (primary); univariate data, heterogeneity, prediction intervals, sensitivity and subgroup analyses also performed
**RESULTS**			
Included studies	7	Give the total number of included studies and participants and summarise relevant characteristics of studies.	Yes – 39 studies (12 153 patients; mostly East Asian retrospective cohorts)
Synthesis of results	8	Present results for main outcomes, preferably indicating the number of included studies and participants for each. If meta-analysis was done, report the summary estimate and confidence/credible interval. If comparing groups, indicate the direction of the effect (i.e. which group is favoured).	Yes – Elevated NLR associated with worse OS (HR 1.51, 95% CI 1.32–1.73), DSS (HR 2.00, 1.58–2.54) and DFS (HR 1.47, 1.21–1.78). PLR and LMR weaker/less consistent
**DISCUSSION**			
Limitations of evidence	9	Provide a brief summary of the limitations of the evidence included in the review (e.g. study risk of bias, inconsistency and imprecision).	Yes – Predominant retrospective design, geographic concentration and variable cut-offs
Interpretation	10	Provide a general interpretation of the results and important implications.	Yes – NLR is a reproducible, inexpensive prognostic biomarker that may complement TNM staging; standardised thresholds require prospective validation
**OTHER**			
Funding	11	Specify the primary source of funding for the review.	Yes – No specific funding was received

Completed for the rewritten abstract (177 words) of the OSCC inflammatory markers systematic review

Table S3

PRISMA 2020 Checklist

Completed for the systematic review and meta-analysis of preoperative NLR, PLR and LMR in oral squamous cell carcinoma

Section and Topic	Item #	Checklist item	Location where item is reported
**TITLE**			
Title	1	Identify the report as a systematic review.	Title page / Title (explicitly states "a systematic review and meta-analysis")
**ABSTRACT**			
Abstract	2	See the PRISMA 2020 for Abstracts checklist.	Abstract section (structured abstract provided)
**INTRODUCTION**			
Rationale	3	Describe the rationale for the review in the context of existing knowledge.	Section 1. Introduction (paragraphs on OSCC epidemiology, TNM limitations, systemic inflammation, and evidence gaps)
Objectives	4	Provide an explicit statement of the objective(s) or question(s) the review addresses.	End of Section 1. Introduction (explicit aims regarding NLR, PLR, LMR and OS/DSS/DFS)
**METHODS**			
Eligibility criteria	5	Specify the inclusion and exclusion criteria for the review and how studies were grouped for the syntheses.	Section 4.2. Selection process
Information sources	6	Specify all databases, registers, websites, organisations, reference lists and other sources searched or consulted to identify studies. Specify the date when each source was last searched or consulted.	Section 4.1. Information sources and search strategy (PubMed/MEDLINE, Scopus, Base, Google Scholar, Science Direct; to 30 April 2026)
Search strategy	7	Present the full search strategies for all databases, registers and websites, including any filters and limits used.	Supplementary Table S1 (complete search strategies for each database)
Selection process	8	Specify the methods used to decide whether a study met the inclusion criteria of the review, including how many reviewers screened each record and each report retrieved, whether they worked independently, and if applicable, details of automation tools used in the process.	Section 4.2. Selection process (two independent reviewers; consensus + senior reviewer; PRISMA flow diagram – Figure 1)
Data collection process	9	Specify the methods used to collect data from reports, including how many reviewers collected data from each report, whether they worked independently, any processes for obtaining or confirming data from study investigators, and if applicable, details of automation tools used in the process.	Section 4.3. Statistical Methods (and implied in Results data extraction for HRs)
Data items	10a	List and define all outcomes for which data were sought. Specify whether all results that were compatible with each outcome domain in each study were sought (e.g. for all measures, time points, analyses), and if not, the methods used to decide which results to collect.	Section 4.3 + Results 2.1–2.3 (OS, DSS, DFS; primary = multivariate HRs; secondary = univariate)

	10b	List and define all other variables for which data were sought (e.g. participant and intervention characteristics, funding sources). Describe any assumptions made about any missing or unclear information.	Table 1 (study characteristics, cut-offs, confounders, demographics, subsites)
Study risk of bias assessment	11	Specify the methods used to assess risk of bias in the included studies, including details of the tool(s) used, how many reviewers assessed each study and whether they worked independently, and if applicable, details of automation tools used in the process.	Section 4.4 (QUIPS tool; results in Figure for RoB domains)
Effect measures	12	Specify for each outcome the effect measure(s) (e.g. risk ratio, mean difference) used in the synthesis or presentation of results.	Section 4.3 (Hazard ratios – HRs)
Synthesis methods	13a	Describe the processes used to decide which studies were eligible for each synthesis (e.g. tabulating the study intervention characteristics and comparing against the planned groups for each synthesis (item #5)).	Results subsections 2.1–2.3 (by marker and endpoint; availability of multivariate vs univariate HRs)
	13b	Describe any methods required to prepare the data for presentation or synthesis, such as handling of missing summary statistics, or data conversions.	Section 4.3 (HR inversion for consistent direction; Tierney method for missing univariate HRs)
	13c	Describe any methods used to tabulate or visually display results of individual studies and syntheses.	Section 4.3 + Results (forest plots, Tables 1–3, scatter plots, funnel plots)
	13d	Describe any methods used to synthesize results and provide a rationale for the choice(s). If meta-analysis was performed, describe the model(s), method(s) to identify the presence and extent of statistical heterogeneity, and software package(s) used.	Section 4.3 (DerSimonian–Laird random-effects + Knapp–Hartung; I^2/tau; prediction intervals; R 4.4.2 with meta/metafor)
	13e	Describe any methods used to explore possible causes of heterogeneity among study results (e.g. subgroup analysis, meta-regression).	Section 4.3 + Results (subgroup analyses by year, area, age, confounders, etc.; cut-off threshold analyses)
	13f	Describe any sensitivity analyses conducted to assess robustness of the synthesized results.	Section 4.3 + Results (leave-one-out sensitivity analyses; cumulative meta-analyses)
Reporting bias assessment	14	Describe any methods used to assess risk of bias due to missing results in a synthesis (arising from reporting biases).	Section 4.3 (contour-enhanced funnel plots, Egger's test, trim-and-fill, Copas selection model for analyses with ≥10 studies)
Certainty assessment	15	Describe any methods used to assess certainty (or confidence) in the body of evidence for an outcome.	Section 4.3 + Results (95% prediction intervals used as primary indicator of robustness; no formal GRADE applied)
**RESULTS**			
Study selection	16a	Describe the results of the search and selection process, from the number of records identified in the search to the number of studies included in the review, ideally using a flow diagram.	Section 4.4 + Figure 1 (PRISMA 2020 flow diagram; 1407 records → 39 studies)
	16b	Cite studies that might appear to meet the inclusion criteria, but which were excluded, and explain why they were excluded.	Section 4.2 (reasons systematically recorded during full-text assessment)
Study characteristics	17	Cite each included study and present its characteristics.	Table 1 (all 39 studies with detailed characteristics)
Risk of bias in studies	18	Present assessments of risk of bias for each included study.	Section 4.4 + associated RoB figure (QUIPS domains; no study rated high risk overall)

Results of individual studies	19	For all outcomes, present, for each study: (a) summary statistics for each group (where appropriate) and (b) an effect estimate and its precision (e.g. confidence/credible interval), ideally using structured tables or plots.	Results 2.1–2.3 + forest plots (Figures 2, 6, 10, 11, 12, 13, 15, 16 etc.)
Results of syntheses	20a	For each synthesis, briefly summarise the characteristics and risk of bias among contributing studies.	Results subsections 2.1.1, 2.1.2, 2.1.3, 2.2, 2.3 (patient numbers, geography, follow-up, confounders)
	20b	Present results of all statistical syntheses conducted. If meta-analysis was done, present for each the summary estimate and its precision (e.g. confidence/credible interval) and measures of statistical heterogeneity. If comparing groups, describe the direction of the effect.	Results 2.1–2.3 + forest plots (pooled HRs, 95% CIs, I^2, tau, 95% prediction intervals)
	20c	Present results of all investigations of possible causes of heterogeneity among study results.	Table 2 (subgroup analyses) + Figure 5 (cut-off scatter plot) + related text
	20d	Present results of all sensitivity analyses conducted to assess the robustness of the synthesized results.	Figures 3, 7, 14 (cumulative and leave-one-out analyses with prediction intervals)
Reporting biases	21	Present assessments of risk of bias due to missing results (arising from reporting biases) for each synthesis assessed.	Results (e.g., Figure 4 for NLR–OS: funnel plot, Egger test, trim-and-fill and Copas adjustments)
Certainty of evidence	22	Present assessments of certainty (or confidence) in the body of evidence for each outcome assessed.	Results + Discussion (primarily via 95% prediction intervals and sensitivity/robustness analyses)
**DISCUSSION**			
Discussion	23a	Provide a general interpretation of the results in the context of other evidence.	Section 3. Discussion
	23b	Discuss any limitations of the evidence included in the review.	Strengths and Limitations subsection of Discussion
	23c	Discuss any limitations of the review processes used.	Strengths and Limitations subsection of Discussion
	23d	Discuss implications of the results for practice, policy, and future research.	Discussion + Conclusion
**OTHER INFORMATION**			
Registration and protocol	24a	Provide registration information for the review, including register name and registration number, or state that the review was not registered.	Section 4.1 (prospectively registered in PROSPERO)
	24b	Indicate where the review protocol can be accessed, or state that a protocol was not prepared.	Section 4.1 (via PROSPERO registration)
	24c	Describe and explain any amendments to information provided at registration or in the protocol.	Not explicitly reported (no major amendments described)
Support	25	Describe sources of financial or non-financial support for the review, and the role of the funders or sponsors in the review.	Funding section ("The authors received no specific funding for this article.")
Competing interests	26	Declare any competing interests of review authors.	Conflicts of Interest section ("The authors declare no conflicts of interest.")
Availability of data, code and other materials	27	Report which of the following are publicly available and where they can be found: template data collection forms; data extracted from included studies; data used for all analyses; analytic code; any other materials used in the review.	Data Availability Statement ("The original contributions presented in this study are included in the article. Further inquiries can be directed to the corresponding author.")
